# Long-read sequencing-based analyses of the adult *Drosophila* brain transcriptome in physiological and pathological settings

**DOI:** 10.1186/s12864-025-12111-w

**Published:** 2025-10-14

**Authors:** Paulino Ramirez, Gabrielle Zuniga, Elizabeth Ochoa, Bess Frost

**Affiliations:** 1 Center for Alzheimer’s Disease Research, Brown University, 185 Meeting St., rm 372, Providence, RI 02912 USA; 2 Barshop Institute for Longevity and Aging Studies, San Antonio, TX 78229 USA; 3 Glenn Biggs Institute for Alzheimer’s and Neurodegenerative Diseases, San Antonio, TX 78229 USA; 4 Department of Cell Systems and Anatomy, University of Texas Health San Antonio, San Antonio, TX 78229 USA

**Keywords:** Long-read sequencing, RNA, *Drosophila*, Transposable elements, RNA methylation, Polyadenylation, Tauopathy, Alzheimer’s disease

## Abstract

Optimal brain function requires that neurons carry out extensive post-transcriptional RNA processing to produce a vast diversity of transcripts. Accurate reconstruction and quantification of highly processed RNA using standard RNA sequencing approaches is challenging due to their short read lengths. Long-read direct RNA sequencing can resolve multiple variations within RNA isoforms by capturing full-length transcripts spanning multiple exon-exon junctions, repetitive regions (e.g. retrotransposons), and intronic structures. Here we produce an isoform-level map of N6-methyladenosine (m^6^A) RNA modifications using Oxford Nanopore Technologies (ONT) long-read sequencing of native RNA strands extracted from heads of *Drosophila melanogaster* aged to day 10 of adulthood. In addition to identifying 930 transcripts that are not present in the reference transcriptome, we find that almost half of the total detected isoforms have polyadenylated tails in excess of 104 nucleotides and that over 59% of transcripts possessed detectable m^6^A-modified bases. RNA modifications are present in RNA transcribed from transposable elements, which are important drivers of genetic diversity and relevant to human neurodegenerative diseases, including Alzheimer’s disease and related tauopathies. Applying nanopore direct RNA sequencing to a *Drosophila* model of tauopathy with known transposable element activation and various types of errors in RNA handling reveals exceptionally diverse RNA processing events in regions that are considered difficult to characterize with traditional short-read sequencing. Taken together, we have uncovered complex transcript structures in adult *Drosophila* head in a physiological setting and in the context of tauopathy, laying the groundwork for future studies to characterize the diverse tau transcriptome in brain tissue from patients with Alzheimer’s disease and related tauopathies.

**Supplementary Information:**

The online version contains supplementary material available at 10.1186/s12864-025-12111-w.

## Introduction

Post-transcriptional regulation is fundamental to gene expression, the process by which DNA is transcribed to RNA to make protein. RNA can undergo a staggering number of post-transcriptional processing steps, each with profound effects on protein production. Most transcripts undergo 5’ capping by a 7-methylguanosine, splicing out of introns and splicing in/out of exons by the RNA itself or by the spliceosome, and polyadenylation, the addition of numerous adenine bases to the 3’ end of the transcript [1]. While the joining of different combinations of exons and introns, known as alternative splicing, generates the greatest diversity in the transcriptome [2], usage of alternative polyadenylation sites also significantly contributes to transcriptome diversity [3]. Transcripts from the same gene can be modified or edited at multiple sites in the 5’ or 3’ untranslated region (UTR), which can affect miRNA binding sites [4] and coding sequences, thus affecting protein production [5, 6].

Neurons are complex cells that have evolved heightened post-transcriptional processing to generate a large number of transcripts from a fixed number of genes. Compared to other cell types, neurons have the highest number of splicing [7, 8] and alternative polyadenylation events [9, 10], and the highest abundance of methylated RNA [11] and transposable element transcripts [12, 13]. The presence of aberrant RNA foci in brain of *Drosophila melanogaster* [14, 15] and human tauopathy [16, 17], a group of neurodegenerative diseases characterized by accumulation of abnormal tau, suggest a mechanistic link between aberrant post-transcriptional RNA processing and neurodegeneration. Efforts to identify error-containing RNA transcripts that may contribute to pathogenicity in neurodegenerative disease are challenging given the extremely dynamic transcriptome changes during neuronal remodeling and rewiring after loss of neurons.

While standard 50–150 bp sequencing methods used for transcript quantification can provide isoform-level expression data, identification of complex isoforms is hindered by shared exons and exon-exon junctions that produce multi-mapping ambiguous reads. Even the most advanced algorithms designed for 50–150 bp sequencing analyses are incapable of fully resolving and quantifying complex, repetitive and/or long RNA isoforms. Long-read sequencing has the potential to address many of these obstacles. This technology utilizes nanoscale protein pores to sense changes in ionic current as negatively-charged, single-stranded RNA transcripts translocate through the nanopore. Motor proteins unwind RNA–DNA duplexes and control the speed of translocation, allowing computational algorithms time to decode changes in the ionic current to a nucleotide sequence in real time [18]. ONT enables sequencing of full transcripts, thus negating the need to assemble smaller pieces of transcripts together using computational approaches. Information on base modifications can also be retained with ONT direct RNA sequencing, as transcripts are not PCR amplified or subject to reverse transcription into cDNA during library preparation. The flow of ionic currents through the protein biosensors that make up ONT nanopores [18] allow for the simultaneous detection of bases and base modifications at near single-nucleotide resolution. Modified RNA bases cause changes in the ionic current that are distinct from unmodified bases, resulting in a unique electrical signal that can be used to determine the presence and position of RNA modifications [18] within each unique transcript.

An additional advantage of long-read sequencing is the ability to better align sequences to complex repetitive regions of the genome [19–24]. Retrotransposons, for example, are abundant and highly repetitive DNA elements that utilize a copy and paste mechanism to mobilize from one location in the genome to another using reverse transcriptase and an RNA intermediate [25]. Retrotransposons are often excluded by standard RNA sequencing analyses due to their repetitive nature and the technical limitations associated with aligning reads of 50–150 bases. This issue is further exacerbated when considering intron retention, splicing events, and epigenetic modifications associated with retrotransposon transcript quantification [22].

Here we analyze RNA extracted from adult *Drosophila* heads, which is predominantly composed of neurons [26], using direct ONT long-read sequencing to identify transcript isoforms and post-transcriptional modifications that are not represented in the latest reference transcriptomes, as well as specific source loci of retrotransposon-derived RNA. As expected, we find that direct RNA long-read sequencing consistently predicts more known transcripts per gene and captures more transcripts containing multiple exons or retained introns than short-read sequencing. We produce an isoform-level transcriptome-wide m^6^A and polyA tail profiles in *Drosophila* heads. Applying nanopore direct RNA sequencing in a *Drosophila* model of tauopathy that expresses abundant transposable elements in the brain reveals the complex splicing, polyadenylation, and methylation profiles that result from pan-neuronal expression of pathogenic tau. Altogether, this dataset generated from a single long-read sequencing approach will be an accessible, easy to use resource for evaluating the epitranscriptome in the adult *Drosophila* head in a physiological setting and in the context of tauopathy.

## Methods

### *Drosophila* genetics

For *Drosophila melanogaster* experiments, an equal number of male and female flies were used. All crosses and aging were performed at 25°C with a 12 h light/dark cycle on standard Bloomington formulation food. The genotype of control flies was *elav-GAL4/* +. The *elav-GAL4* driver stock (Bloomington stock number 458) and *w*^*1118*^ background stock (Bloomington stock number 5905) were obtained from the Bloomington *Drosophila* Stock Center. Dr. Mel Feany kindly provided the UAS-tau^R406W^ flies [27]. Henceforth, R406W mutant tau (tau^R406W^) is referred to as “tau” for simplicity. We used the panneuronal *elav* promoter and the GAL4-*UAS* system to produce transgenic tau^R406W^ *Drosophila*.

### RNA isolation and sequencing

#### Nanopore long-read sequencing

Total RNA was extracted from 500 10-day-old adult *Drosophila* heads using TRIzol (Invitrogen, 15,596,026); RNA concentrations were measured on a Nanodrop 8000 spectrophotometer. Total RNA isolated from *Drosophila* heads was analyzed via RNA ScreenTape prior to ONT library generation; all samples achieved an RIN > 9.8. 75 μg of total RNA was enriched for full-length polyA RNA using the Dynabeads mRNA Purification kit (Invitrogen, 61,005); 500 ng polyA tailed RNA was used for library preparation using the Direct RNA Sequencing kit (SQK-RNA002) and protocol from ONT [28]. RNA quality of the Libraries was assessed using a Qubit 4 fluorometer (Invitrogen) prior to sequencing using the MinION Mk1C with R9.4 flow cells. Three biological replicates per genotype was generated.

#### Illumina short-read sequencing

Total isolated RNA was extracted as previously described [29] for six biologically independent replicates per Genotype. Briefly, 10 fly heads (five female and five male) per biological replicate were homogenized in radioimmunoprecipitation buffer (50 mM tris–HCL, 0.5% Nonidet-P40, 1.5 mM MgCl, 140 nM NaCl, and 1 mM dithiothreitol) with 1 μL (40 units/μL) of RNaseOUT (Invitrogen) and precleared with 40 μl of protein A Dynabeads (Invitrogen) for one hour at 4°C. After bead clearance, precleared lysate was split in two aliquots and diluted to a volume of 200 μl with cold binding buffer (0.0025% Triton X-100 in 1 × PBS). Precleared lysate (200 μl) was stored at − 80°C for RNA extraction via TRIzol. All RNA concentrations were measured by Qubit and RNA quality was assessed via High Sensitivity RNA ScreenTape on an Agilent TapeStation.

RNA Libraries from 0.7–1 μg of RNA were generated using the TruSeq Stranded mRNA Library Prep Kit and accompanying Index Set (Illumina Inc). Following quality control via High Sensitivity DNA ScreenTape (Agilent), pooled libraries were sequenced on the Illumina HiSeq3000 platform via 150 bp paired-end sequencing. Quality control and RNA sequencing was performed by the Genome Sequencing Facility at the Greehey Children's Cancer Research Institute at the University of Texas Health San Antonio.

### Basecalling and quality control of long reads

Basecalling was performed with Guppy (v5.1.12) [30]. Reads with a quality value of 9 or more were used for further analysis and aligned to the *Drosophila melanogaster* (FlyBase Dmel 6.43) genome or transcriptome with minimap2 [31] utilizing suggested parameters for nanopore direct RNA sequencing files. Sequencing metrics and alignment statistics were generated with SAMtools [32] and BamSlam [33]. Full length reads identified by BamSlam [33] are defined as reads with 95% or more coverage of a known transcript. Full reads were further defined as a read whose 5’ end aligns with a known transcription start site (TSS) and whose 3’ end aligns with a known polyadenylation site (PAS) from the same reference transcript, as defined by FlyBase (GTF Dmel 6.43) using the GenomicFeatures and GenomicAlignments [34] packages. Reads whose ends overlapped both sites for a single isoform within 200 bp were considered a high-quality full-length read. Quality control plots were generated from Nanoplot [35]. To determine if a more accurate model would result in significant changes in splicing, we recalled bases with Dorado (v0.9.6), using default parameters. Guppy-derived basecalls were utilized for all analyses unless stated otherwise.

### Genome-guided transcriptome assembly

The three control ONT long-read sequencing libraries were combined for genome-guided transcriptome assembly. Pooled sequencing files were aligned to the *Dmel* 6.43 genome using minimap2 [31] with disabled secondary mapping (-ax splice -uf -k14 -t 12 –secondary = no). StringTie (v2.2.1) [36] was used to generate a transcriptome-guided assembly of known/non-reference transcripts and transposable elements, which are not included in the FlyBase annotated transcriptome. To obtain a de novo transcriptome that includes transposable elements, we supplemented the *Dmel* 6.43 genome annotation with TEtranscripts-based *Drosophila melanogaster* transposable element annotation [37]. Isoforms were included in the final StringTie-assisted de novo transcriptome if they were supported by at least one read. This process was then repeated using the six sequencing libraries obtained from Illumina sequencing. For Illumina-based assembly, the reads were aligned with Bowtie2 [38] to the *Dmel* 6.43 genome. ONT and Illumina transcriptome assemblies were compared to the FlyBase annotated transcriptome using Structural and Quality Annotation of Novel Transcript Isoforms (SQANTI) [39] to identify non-reference isoform and intergenic transcripts. This process was then repeated for pooled tau samples. Correlation analysis for ONT vs. Illumina and pooled control vs. pooled tau were computed with a Pearson correlation analysis using the ggstatsplot [40] R package.

### Differential expression analysis in control vs. tau transgenic *Drosophila*

#### Reference-independent method

FLAIR (v1.5) [41] reference-guided transcriptome containing both previously-annotated and non-annotated transcripts, as well as transposable element isoforms lacking in the FlyBase annotation. Unlike typical de novo transcriptome assembly, FLAIR relies on genome annotations (*Dmel* 6.43 supplemented with TEtranscripts *Drosophila melanogaster* transposable element annotation [37]) rather than the transcriptome itself. This approach allows for identification of transposon-derived transcripts at the loci and family levels.

To obtain the highest quality transcriptome, all six library-specific transcriptomes were collapsed into a single transcriptome for further analysis, as suggested by the developers [41]. Isoforms supported by at least three reads (spanning 80% of the isoform) from any sequenced sample were included in the final transcriptome. The FLAIR de novo transcriptome underwent filtering with SQANTI3 [42] to remove false positive novel isoform transcripts that were Likely a result of 3’ sequencing bias or degraded transcripts due to sample freeze–thaw. The filtered final transcriptome was manually curated to ensure that previously annotated FlyBase-derived isoforms were not discarded as artifacts. The resulting reference-guided transcriptome was used for all splicing analyses. Isoforms were categorized using a combination of SQANTI [39] and FLAIR (premature termination codon (PTC))-derived annotations; abundance of each isoform was determined using the FLAIR quantify module.

Abundance of isoforms belonging to SQANTI structural categories were analyzed in control vs. tau transgenic *Drosophila*. FLAIR isoform counts were normalized using upper quantile normalization in edgeR [43]. Isoform fold-changes were determined by dividing the median expression isoform counts of tau transgenic *Drosophila* by controls. The distribution of isoform fold-change expression between isoform categories such as mono-exon, alternative transcription start site, alternative transcription termination site, novel combination of known reference, and intron retention isoforms were compared to the reference match isoform category via two sided Mann–Whitney U test. FLAIR identified PTC-containing isoforms were then compared to “normal,” errorless isoforms.

#### Reference-guided method

The NanoCount [44] pipeline was used to identify differentially expressed transcripts between control and tau transgenic *Drosophila* that are included in the FlyBase reference genome. Reads were aligned to the *Dmel* 6.43 transcriptome with minimap2 (-ax map-ont -p 0 -N 10). Isoform abundance counts were based on NanoCount (V1.0.0 default parameters); differential isoform expression was subsequently analyzed with DESeq2 [45].

### Analysis of polyA tail length

PolyA tail lengths of individual isoforms included in the FlyBase annotated transcriptome were quantified using nanopolish [46] (https://github.com/nanoporetech/pipeline-poly(A)-diff). PolyA tail lengths were further classified as short (< 64 nt), medium (> 64 and < 82 nt), or long (> 82 nt), based on their interquartile values of the distribution of median polyA tail length of control samples. Interquartile values were then calculated for tau samples. Isoforms with at least five polyA tail length measurements per sample were considered in differential polyA tail length analysis of control vs. tau transgenic *Drosophila*. Differential polyA tail lengths were identified by comparing median polyA tail length per isoform via a Mann–Whitney U test with multiple testing correction. Isoforms with a false discovery rate (FDR) of 0.05 or less were considered significantly different. PolyA tails for sample control three were quantified using tailfindr [47] (default settings) as a secondary method of analysis. Pearson’s correlation analysis was then performed to determine the congruence of nanopolish-derived vs. tailfindr-derived polyA tail length.

### Analysis of m^6^A modification

The m6Anet pipeline [48] was used identify consensus modification rates of nucleotides within transcripts (*Dmel* 6.43 reference isoforms or FlyBase consensus transposons) in individual and Pooled control and Pooled tau samples. Modification call sites with a minimum probability of 0.9 were considered for further analysis. Thresholds for “low, medium and high” were set by calculating the relative 25th and 75th percentiles of m^6^A modification. Values below the 25th percentile are classified as low, values from 25 to 75th percentile are classified as medium, and values greater than the 75th percentile are classified as high. Differential N6-methyladenosine (m^6^A) modifications of mRNA isoforms or transposable elements (FLAIR isoforms as identified above) in tau transgenic *Drosophila* compared to controls were analyzed using xPore (v2.1) [49] with recommended parameters. Calls with an absolute modification level change of at least 0.4 and an FDR of 0.05 or less were considered significant. xPore output was then filtered to retain only DRACH modifications. Hypermethylated and hypomethylated sites were defined as modification rates with a greater than or less than, respectively, the modification rate of the matching control site. Only sites with a modification call in each sample were used for analysis. m^6^A sites with an FDR less than 0.05 were considered significant. Meta-genomic transcripts were created using the R package GenomicPlots [50] with default parameters, using m^6^A motif bed files created from m6Anet. Genomics features were gathered from the R package TxDb.Dmelanogaster.UCSC.dm6.ensGene [51]. To compare our results to published data [52], we then plotted miCLIP-identified m^6^A sites of 3-day old *Drosophila* heads along the meta Gene and determined the overlap within 1,000 bp of the miCLIP sites and peaks with the m6Anet sites with the GenomicRanges R package [34]. Additionally, we determined the overlap of m6A-containing gene transcripts between m6Anet and genes identified in Sami et al.

A meta-copia RNA transcript was computationally generated based on an m6Anet analysis of pooled control fly data using m^6^A calls on full-length *copia* sequences from FlyBase and visualized as a segment graph. The x-axis of the graph represents each nucleotide of the *copia* transcript in its entire length (0 to 5,186 nucleotides). An m^6^A call was plotted on its respective nucleotide if detected on any full-length *copia* sequence. The functional *copia* gene body and Long Terminal Repeats (LTRs) were subsequently added by manually annotating the full-length *copia* sequence.

### Gene ontology analysis

Genes associated with post-transcriptional modifications (i.e. alternative splicing, polyA tailing, methylation) were selected for enrichment analysis to determine biologically significant differences between control and tau transgenic *Drosophila* in splicing, polyA tail length, and m^6^A site frequency. Gene ontology annotation was performed with ShinyGO [53] and the R package clusterProfiler [54]. Parent genes were used in place of isoforms, as isoforms do not currently have associated ontology terms and were used as a background gene list. Fisher’s exact test (FDR < 0.05) followed by the Benjamini–Hochberg procedure was used to determine significantly enriched terms. Gene ratio is defined as the number of query genes with the associated GO term divided by the total analyzed gene query.

The code for all steps used to create transcriptomes and figures is available at: 10.5281/zenodo.15603223.

## Results

### Direct long-read RNA sequencing of transcripts isolated from adult *Drosophila* heads

The relatively small (~ 180 Mb) *Drosophila* genome has been extensively studied and is one of the best annotated genomes to date [55–58]. Our selection of nanopore direct RNA sequencing was driven largely by the ability to obtain long reads and accompanying epitranscriptome data. To ensure the data generated by nanopore direct RNA sequencing was of high quality, we first gathered metrics on sequencing performance in control flies (Table 1). Sequencing was performed on polyA-enriched RNA extracted from heads of *Drosophila* at day 10 of adulthood to a depth of approximately one million reads (max of 1.25 million reads) per sample. The median read quality is similar among samples. The average length of aligned reads is 1,363 nt, similar to results from cDNA-derived reads of 1–2 kbp in non-size selected assays [59]. 91–92% of reads map to the *Dmel* reference genome; 33–43% of reads align completely to full-length reference transcripts present in the FlyBase reference transcriptome (90% or more match to isoform). When setting more stringent standards requiring a read cover both the TSS and PAS site of a single isoform, approximately 3% of reads are classified as full length.

**Table 1:** Nanopore direct RNA sequencing quality metrics of control flies

	**Control 1**	**Control 2**	**Control 3**
Total reads sequenced	1,251,199	1,013,093	771,801
Longest isoform sequenced (nt)	29,482	20,019	14,356
Median read quality	11.4	11.9	11.5
Read length (n50)	1,297 nt	1,439 nt	1,354 nt
Median fraction of reference transcript covered by each read	0.85	0.92	0.91
Percentage full-length reads	33.8	41.4	40.8
Mapping percentage to *Dmel* genome	91.5	92.0	91.2
TSS/PAS-verified full-length reads	2.94	3.29	3.17

We next analyzed polyA-enriched transcripts identified via Illumina 150 bp sequencing to compare the quality of the de novo transcriptome assemblies based on direct long-read sequencing. While Illumina sequencing produces drastically more reads than ONT (223 million vs. 3 million reads, respectively; Supplemental Table 1), transcript quantification is highly concordant between 150 bp sequencing and ONT (R = 0.73) (Supplemental Fig. 1A). Of note, there is little overlap (two transcripts) between non-reference transcripts detected via ONT vs. Illumina-derived assemblies. The length of transcript isoforms is on average 1,363 nt in the long read assembly (Supplemental Fig. 1B). In line with previous reports [60–62], the de novo transcriptome assembled via direct long-read RNA sequencing without reference genome annotation includes more isoforms than 150 bp Illumina sequencing (Supplemental Table 2).

**Fig. 1:**
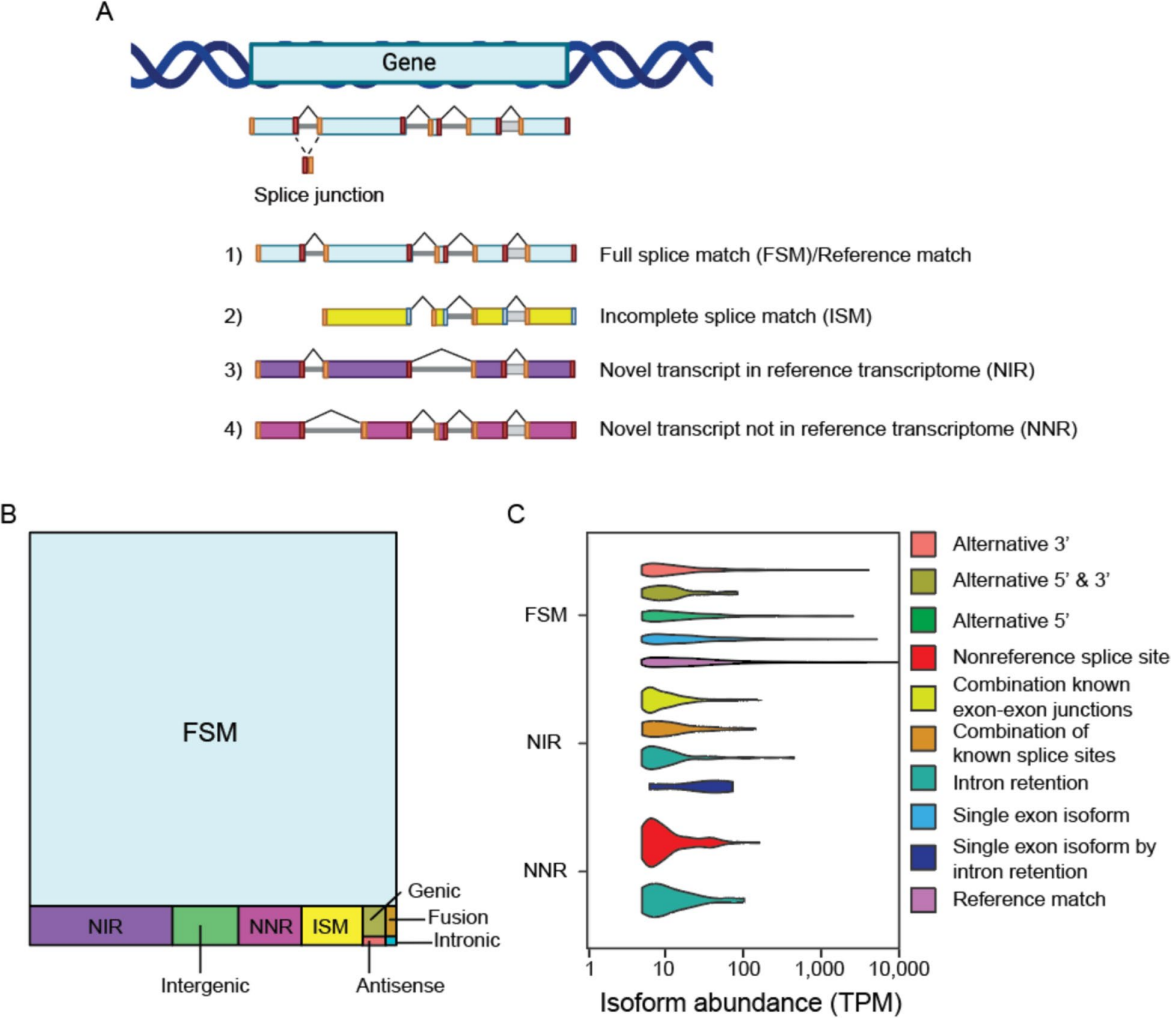
Classification of structural variants detected in the *Drosophila* head transcriptome. **A** Graphic of structural categories detected by SQANTI. **B** Treemap showing proportion of isoform categories in the *Drosophila* head transcriptome. **C** Isoform abundance (transcripts per million (TPM)) for structural categories and subcategories visualized via violin plot. All flies were aged to day 10 of adulthood

### Identification of non-reference splicing variants in *Drosophila* heads

We applied the SQANTI analysis pipeline for in-depth characterization of isoform splicing variants obtained from long-read sequencing of RNA isolated from *Drosophila* heads. SQANTI classifies transcripts into structural categories: 1) Transcripts with the same number and position of splice junctions as the reference transcript are classified as full splice match (FSM), 2) Transcripts matching a reference transcript at all internal splice junctions but missing one or more external exons (5’ or 3’ end exons) are classified as an incomplete splice match (ISM), 3) Novel transcripts using new combinations of previously annotated acceptor and donor splice sites are classified as a novel transcript in reference transcriptome (NIR), and 4) Novel transcripts using combinations of at least one unannotated acceptor or donor site are classified as novel transcripts not in reference transcriptome (NNR) (Fig. 1A). Other novel isoform categories include transcripts that are oriented antisense to an annotated gene, fusion transcripts composed of transcripts from two or more annotated genes, intergenic transcripts that lie outside annotated genes in the reference genome, genic transcripts overlapping with both introns and exons, and intronic transcripts that are contained within a reference intron (Supplemental Fig. 2).

**Fig. 2:**
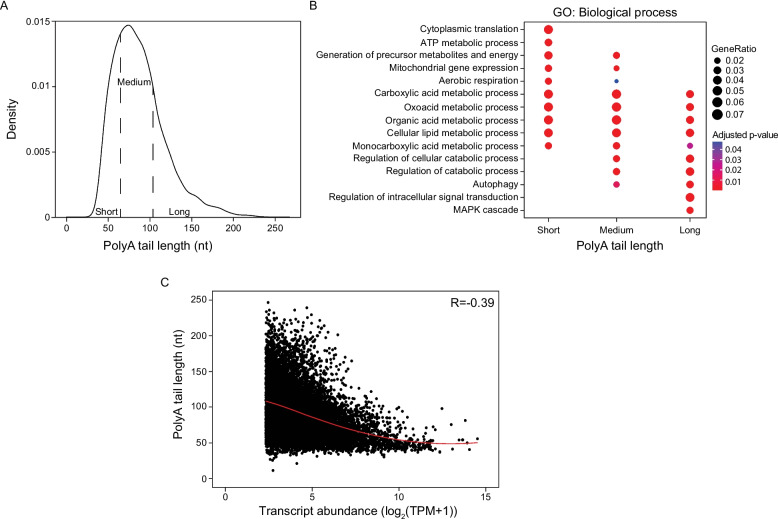
Long-read sequencing-based profiling of polyA tail length in the *Drosophila* head transcriptome.** A** Density plot of individual isoform polyA tail lengths categorized as short (< 64 nt), medium (64–82 nt) or long (> 82 nt).** B** Dotplot for most significantly enriched GO terms among short, medium and long polyA tails. **C** Scatterplot of transcript levels in relation to their corresponding polyA tail lengths, with a polynomial fitted line (red). All flies were aged to day 10 of adulthood. All graphs were made using a median polyA tail length of the control samples

Transcripts that fall into the FSM and ISM category account for 91.6% and 1.51%, respectively, of transcript types sequenced from *Drosophila* heads using nanopore direct RNA sequencing (Fig. 1B). FSM transcripts tend to be longer (Supplemental Fig. 3A) than their annotated length indicated in the FlyBase reference transcriptome, suggesting that long-read sequencing can more accurately capture FSM transcripts with large variation at the 3’ and 5’ ends. We find that transcript isoforms with alternative 3’ ends tend to be longer than their reference transcript (Supplemental Fig. 3A), suggesting that our long-read sequencing data may be biased toward the 3’ end of transcripts due to fragmentation and pore blocking, and that long transcripts (> 1,000 nt) are particularly at risk. Isoforms categorized as novel transcripts in reference transcriptome, novel transcripts not in reference transcriptome, intergenic, Genic, fusion, and antisense transcripts account for 3.7%, 1.57%, 1.59%, 0.46%, 0.22% and 0.1%, respectively, of the adult *Drosophila* head transcriptome (Supplemental Table 2). Novel antisense and intronic transcripts mapping to the reference transcriptome are negligible. Compared to 150 bp Illumina reads, long reads have greater power to detect NIR transcripts and filter out genic fragments that may result from RNA degradation vs. ambiguous mapping (Supplemental Fig. 3B).

**Fig. 3:**
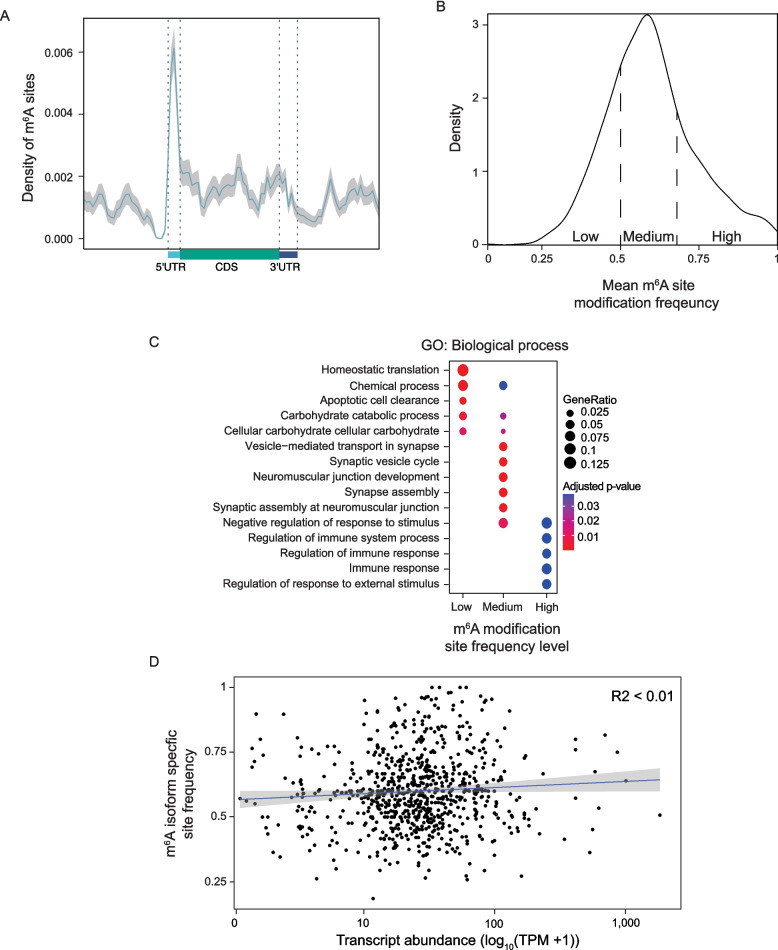
Isoform-level analysis of RNA methylation in the *Drosophila* head transcriptome.** A** Density plot of m^6^A site enrichment across a meta gene from the 5’ to 3’ end (spanning the 5’ UTR, coding sequence, and 3’ UTR) from the pooled control samples. The gray line indicates the 95% confidence interval. **B** Density plot of mean m^6^A modification frequency of detected sites in (**A**). High, medium, and low mean m^6^A modification frequency at specific sites defined by 0.17–0.5, 0.5–0.59, and 0.59–1 interquartiles. **C** Dotplot for most significantly enriched GO terms in isoforms with low, medium and high m^6^A site location frequency. **D** Scatterplot of transcript abundance vs. m^6^A isoform site frequency. The linear best fit is indicated by a blue line; the 95% confidence interval is indicated in gray. All flies were aged to day 10 of adulthood. All graphs were made using a pooled control sample set

SQANTI-based analysis of nanopore direct RNA sequencing allows the subclassification of non-reference isoform categories based on the type of splicing changes (Supplemental Fig. 2). We find that most NIR transcripts can be classified as novel combinations of known exon-exon junctions or splice sites, novel introns, and to a lesser extent, the genic subcategory (Fig. 1C). Intron retention is the most abundant alternative splicing event in the NIR category identified via direct long-read RNA sequencing (Fig. 1C), but not 150 bp RNA sequencing (Supplemental Fig. 3C). Consistent with previous studies [14], these data suggest that *Drosophila* neurons increase the diversity of transcripts largely through intron retention.

### Long-read sequencing-based polyA tail length profiling in the adult *Drosophila* head

We next sought to qualitatively and quantitatively characterize post-transcriptional RNA modifications at single base resolution in *Drosophila* heads. PolyA tailing is an RNA modification that involves the rapid addition of 70–250 adenosines, depending on the species [63], to the 3’ end of RNA prior to nuclear export. We confirm that nanopore direct RNA sequencing efficiently detects polyA tails of RNA isoforms up to the critical length of approximately 250 nt [64] (Fig. 2A). The median polyA tail length per isoform in *Drosophila* heads is 82.6 nt, consistent with a median polyA tail length of ~ 80–90 nt based on polyA tail length profiling in mouse visual cortex [65] and 3’ end capture of nanopore cDNA sequencing in mouse brain [66]. These data suggest that the length of polyA tails in the mouse central nervous system is conserved in the *Drosophila* brain.

In most eukaryotes, mRNA with long polyA tails are enriched for genes encoding transcription factors, hormone receptors and various other regulatory proteins, while transcripts with short polyA tails are enriched for essential genes associated with translation and nucleosome complexes [67]. Gene ontology analysis reveals that transcript isoforms with short polyA tails (< 64.45 nt) are enriched for translation and immune processes, while isoforms with long polyA tails (> 103.57 nts) are enriched for metabolic processes and mRNA and protein processing pathways in adult *Drosophila* heads (Fig. 2B). We find that transcripts with short median polyA tail lengths are more abundant in *Drosophila* heads (Fig. 2C), consistent with studies in yeast, *Caenorhabditis elegans*, *Drosophila* S2 cells, and mouse cell lines [67]. We compared polyA tail length estimates from nanopolish to those identified via tailfindr for secondary validation. A Pearson correlation coefficient of 0.99 indicates a high degree of concordance between the two methods (Supplemental Fig. 3D).

### Long-read sequencing-based analysis of m^6^A methylation in the adult *Drosophila* head

Having determined the distribution of isoform-specific polyA tail modifications from base-called nanopore direct RNA sequencing, we next evaluated m^6^A methylation, a highly abundant RNA modification in the brain [11], in full-length native RNA isoforms. m^6^A modification of a known m^6^A modification motif (5’-DRACH-3’) is detected in nanopore direct RNA sequencing data as a distinct shift in current intensity and dwell time through the nanopore [20, 68]. We find that m^6^A modification sites are variable across the transcript with enrichment towards the 5'UTR and transcription start site (Fig. 3A), consistent with m^6^A miCLIP analysis of *Drosophila* heads at day three of adulthood [52] (Supplemental Fig. 3E) and m^6^A RNA immunoprecipitation sequencing (m^6^A RIP-seq)-based analysis of the *Drosophila* larval brain [68] (Supplemental File 1). To verify the m^6^A sites predicted by m6Anet, we compared these results to those obtained using TandemMod, a recently developed detection method capable of identifying multiple types of RNA modifications in eukaryotes [69]. While there was no overlap at the level of individual m⁶A sites, 618 out of 684 transcripts were identified as m⁶A-modified by both tools, indicating a high degree of concordance at the transcript level (Supplemental File 2).

We next performed gene ontology analysis of transcripts based on their mean modification frequency (low: 0.17–0.5, medium: 0.5–0.59, high: 0.59–1.00) to gain insight into functional relationships of variable m^6^A levels across transcripts. The distribution of average m^6^A modification frequency of *Drosophila* transcripts is shifted towards a high level of modification, with a wide range of 0.68 to 1.00 and median frequency of 0.58 (Fig. 3B). Highly modified transcripts are significantly enriched for immune system processes, while transcripts with medium levels of modification are enriched for terms related to synaptic function (Fig. 3C). Transcripts with lower levels of m^6^A are enriched for cellular processes that maintain homeostasis. These analyses support evidence that m^6^A modifications increase functional diversity of transcripts beyond what is determined by the RNA sequence [70]. We fail to identify a correlation between transcript abundance and m^6^A modification rate (Fig. 3D) as reported by others [52, 71]. As m6Anet is reported to yield false positives and exhibit sequence context biases [72], we evaluated its performance by comparing m6Anet m⁶A site predictions to those identified in *Drosophila* heads via miCLIP of *METTL3* knockouts versus control [52]. Of the 711 genomic m⁶A sites identified by m6Anet, 271 (~ 38%) fall within 100 bp of METTL3-dependent m⁶A peaks and high-confidence predicted m⁶A sites (Supplemental File 3). These results suggest that, despite methodological differences, our analyses recapitulate a substantial subset of previously validated m^6^A modification sites.

### Long-read sequencing-based analysis of transposable element-encoded transcripts in the adult *Drosophila* head

**Fig. 4:**
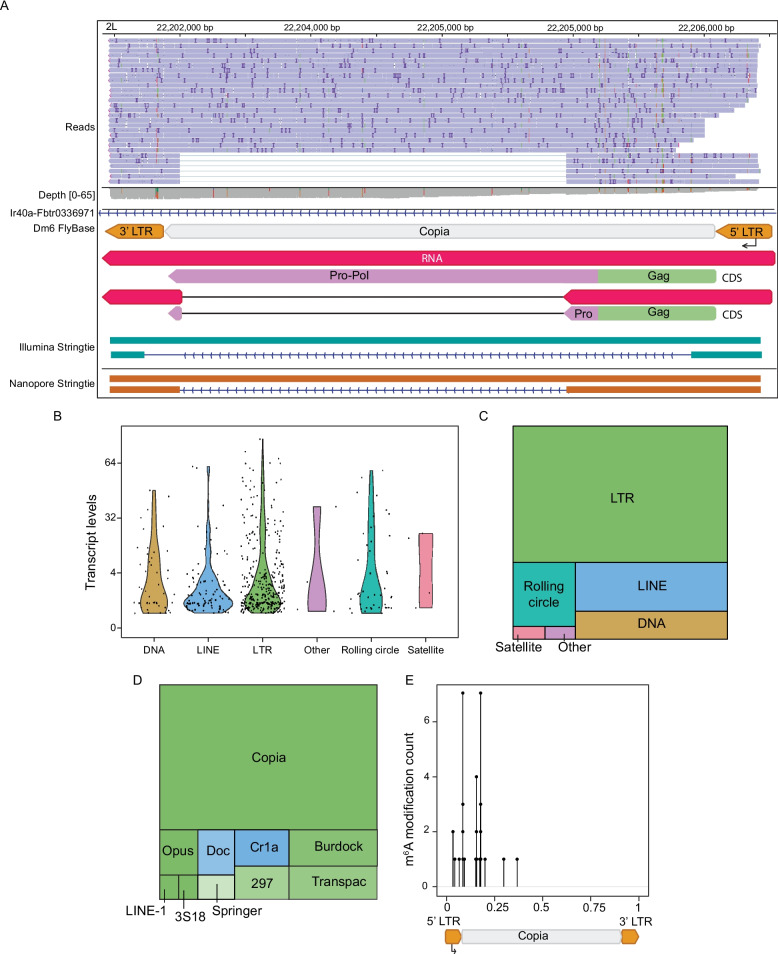
Locus-specific quantification of transposable element-encoded transcripts in the *Drosophila* reference genome.** A** Long- and short-read based analysis of a *copia* element on chromosome 2. IGV visual alignment of long reads to the *copia* element with marked insertions (purple lines), deletions (white space), and nucleotide differences to the reference genome. *Copia* DNA (gray, orange), *copia* RNA (pink), *copia* predicted coding sequence (purple and green), *copia* isoforms assembled from short reads (turquoise), *copia* isoforms assembled from long reads (dark orange), lines with arrows represent introns. **B** Violin plot of transposable element-derived transcripts grouped by family.** C** Treemap of the proportion of transposable elements detected grouped by family. **D** Treemap of m^6^A sites detected on transposable element-derived transcripts grouped by family. **E** Lollipop plot of m^6^A modifications of *copia* transcripts. Locations of m^6^A modifications are normalized to a consensus *copia* element. All flies were aged to day 10 of adulthood. All graphs were made using a pooled control sample set

While transposable elements compose a large fraction of the *Drosophila* genome, their repetitive nature limits the ability of Illumina-based sequencing approaches to identify the source locus from which a given retrotransposon transcript originates. To quantify expression levels from specific retrotransposon source loci, we initially focus on *copia*, a well-studied long-terminal repeat (LTR) retrotransposon in *Drosophila*. Transcript assemblies generated by nanopore direct RNA sequencing long reads, but not Illumina short reads, identify the correct spliced *copia* isoform responsible for production of gag, a protein that is central to viral-like capsid formation [73] (Fig. 4A). Given the improved mapping of long reads to the *copia* repetitive element, we expanded our search to identify source loci for additional elements. While many retrotransposon loci have low expression levels (TPM < 5), and therefore low confidence in whether the identified site is the true source site, others reach levels comparable to *Drosophila* housekeeping genes (actin (FBtr0303048) = 16 TPM) and eEF1α1 (FBgn0284245) = 800 TPM) (Fig. 4B, Supplemental File 4). We find that DNA, long-interspersed nuclear elements (LINE), rolling circle or Helitrons, LTR, and satellite-sequence derived transposons that are often found to be transcriptionally active are highly expressed in heads of tau transgenic *Drosophila*. Among these highly expressed transposable element families, the active LTR transposable elements predominate (Fig. 4C), largely due to the contributions of *copia* transcripts (Supplemental File 4). Transcripts of non-*copia* transposable elements are detected for *BEL-PAO* (*3S18{}853*), *Transpac* (*Transpac{}32*), and *DOC* (*Doc{}CHKov1*^*Doc1420*^) (Supplemental File 4). While m^6^A modifications are present across multiple transposable element subfamilies, m^6^A modifications are particularly enriched on *copia* transcripts, with stronger bias toward the 5' UTR (Fig. 4D, E).

### Nanopore direct RNA long-read sequencing-based analysis of a *Drosophila* model of tauopathy

We next used nanopore direct RNA sequencing to analyze differential transcript expression, polyA tail length, m^6^A modification, and transposable element expression/m^6^A modification in a well-described *Drosophila* model of tauopathy with documented deficits in RNA processing. *Drosophila* with pan-neuronal transgenic expression of human tau carrying frontotemporal dementia-associated R406W mutant tau [74] have extensive evidence of RNA mishandling, including increased intron retention [14], activation of transposable elements [75, 76], alterations in RNA export, and deficits in nonsense-mediated RNA decay [15]. This model features age-dependent neurodegeneration, with a moderate degree of neuronal death by day 10 of adulthood [74].

**Fig. 5:**
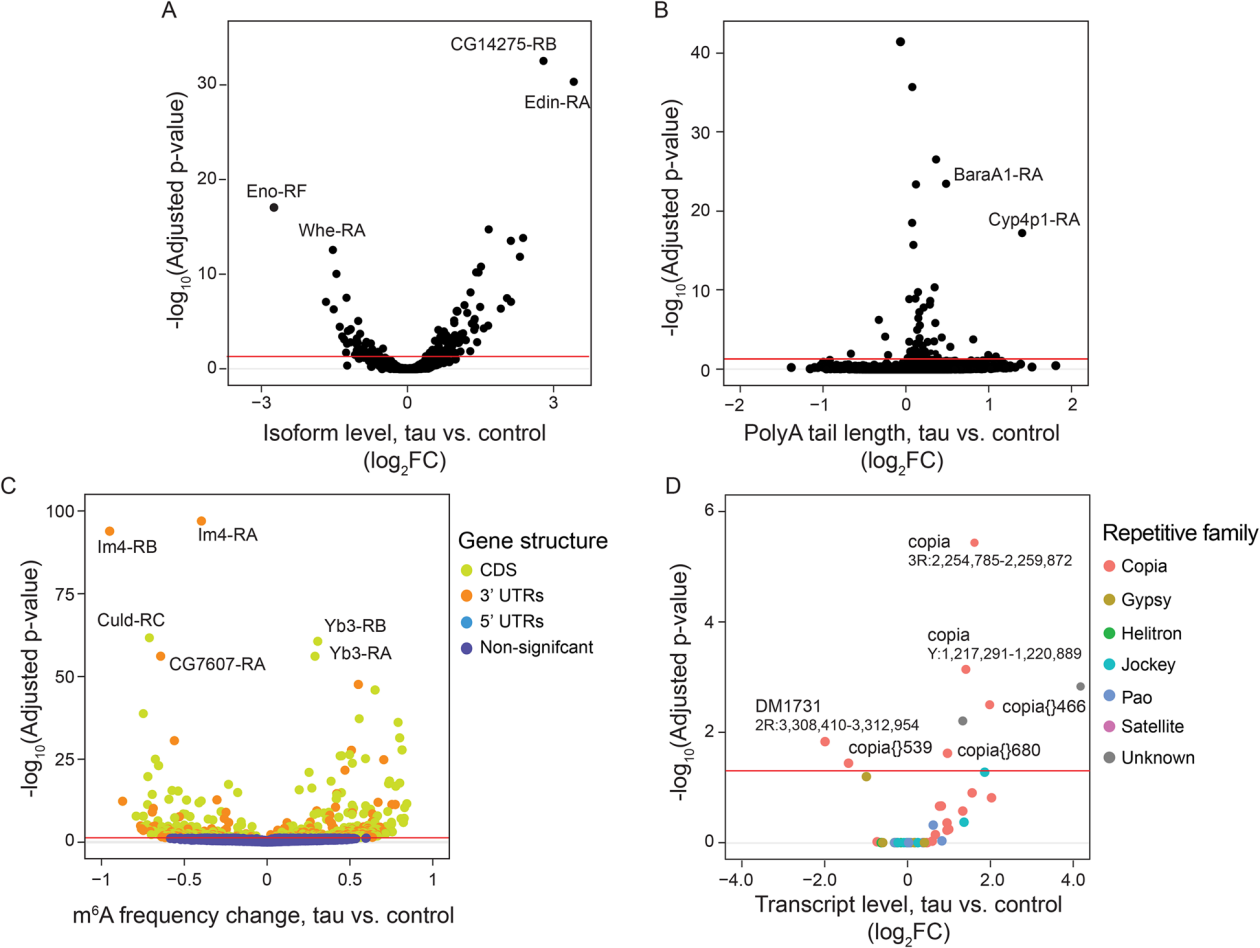
Nanopore direct RNA sequencing-based analysis of the transcriptome in heads of tau transgenic *Drosophila*. Volcano plot representing (**A**) differentially expressed FlyBase transcript isoforms between control and tau transgenic *Drosophila*, (**B**) median polyA tail length of FlyBase transcript isoforms that are differentially expressed between control and tau transgenic *Drosophila*, (**C**) FlyBase transcript isoforms that are differentially m^6^A modified between control and tau transgenic *Drosophila* (largest isoform change is not shown), and **D)** differentially expressed transposable elements in control vs. tau transgenic *Drosophila*. The red line denotes significance (adjusted *p* value < 0.05). All flies were aged to day 10 of adulthood

We enriched for full-length polyadenylated RNA transcripts from heads of *Drosophila* aged to 10 days and sequenced using direct nanopore sequencing (Table 1, Supplemental Table 3). We first identified RNA isoforms in the FlyBase reference transcriptome that are differentially expressed between control (*elav-GAL4/* +) and tau transgenic *Drosophila (elav-GAL4/* + *; UAS-tau*^*R406W*^*/* +*)*. Principal component analysis indicates that samples group by genotype (Supplemental Fig. 4A). To account for potential differences due to sequencing depth and other variables, we performed a Pearson correlation of TPM values of all FlyBase reference transcript TPM values. Samples show high correlation (0.95–0.99), indicating high replicability of sample libraries (Supplemental Fig. 4B). We detect 137 differentially expressed isoforms (adjusted p-value of 0.05 or less) from 119 genes (Fig. 5A), indicating that some genes encode multiple isoforms that are differentially expressed in the tau model. Among genes with multiple differentially expressed isoforms, most isoforms change in the same direction for a given gene. The *ribosomal protein S21* (*RpS21*) gene, which encodes a translation initiation factor upregulated in cancer [77], generates transcripts that are alternatively spliced into two isoforms, one of which is increased in tau transgenic *Drosophila* compared to controls, and another that is decreased in tau transgenic *Drosophila* (Supplemental Fig. 4C). While *enolase*, a neuron-specific enzyme that increases in patients with poor neurological outcomes [78], is not differentially expressed at the gene level, we find that a specific isoform of *enolase* is depleted by approximately three-fold in tau transgenic *Drosophila* compared to controls (Supplemental Fig. 4D**, **Supplemental File 4). The human homolog of *enolase*, *ENO1*, has been nominated as a potential target for Alzheimer’s disease by the Accelerating Medicines Partnership in Alzheimer’s Disease (AMP-AD) consortium.

Unlike previous analyses [14, 79], we do not detect significant changes in global alternative splicing events such as intron retention and novel combinations of splicing sites (“combination of junctions”) due to expression of pathogenic tau (Supplemental Fig. 4E, F), likely reflecting a limitation of the need to pool large numbers of *Drosophila* heads for direct long-read sequencing. To determine if changes could be detected with a larger sequencing depth, we pooled biological replicates and determined changes in alternative splicing events, with a focus on intron retention. A Fisher’s exact test shows that the tau genotype has a significantly higher proportion of intron-containing transcripts compared to control (*p* value = 1.8e-6) (Supplemental Fig. 4G, H). Repeating the splicing analysis with Dorado, a recently developed tool for basecalling [80], shows no significant differences from results obtained with the Guppy basecaller (Supplemental Fig. 4G, H). Analysis of isoform-specific polyA tail length profiles, however, reveal that tail length is modestly extended in tau transgenic *Drosophila* (Fig. 5B; Supplemental File 5, Supplemental Fig. 5A, B). 73 of 79 transcript isoforms with statistically significant differential polyA tail lengths harbor a longer polyA tail in the tau condition. At the single-molecule level, we find that variability of polyA tail detection per read can differ between individual reads (Supplemental Fig. 5C, D). Gene ontology analysis of mRNA with altered polyA tail lengths in tau transgenic *Drosophila* identified genes involved in the immune response and catabolic processes (Supplemental Fig. 5E). Among differentially expressed transcripts with significantly modified polyA tails between tau transgenic *Drosophila* and controls, we detect a negative correlation (*p* value = 0.03) between polyA tail length and isoform expression (Supplemental Fig. 5F). As transgenic human tau is specifically expressed in neurons of the *Drosophila* model used in the current study, we next focused our analysis on isoforms whose genes are known to be expressed in neurons [81]. While lengths of polyA tails do not differ for individual transcripts associated with either neurons or glia, we find that the general class of transcripts associated with neurons have longer polyA tail lengths compared to transcripts associated with glia in both control and tau transgenic *Drosophila* (Supplemental Fig. 5G). Analysis of polyA tail length, m^6^A enrichment, and transcript abundance indicate no significant correlation (*p* value = 0.15) between m^6^A modification rate and polyA tail length in tau transgenic *Drosophila* compared to controls (Supplemental Fig. 5H).

We next investigated tau-induced changes in m^6^A modifications. Of the 8,305 individual m^6^A motifs detected from 4,620 isoform transcripts, we identify 529 transcript isoforms marked with 558 m^6^A motifs that are significantly different (FDR less than or equal to 0.05) in tau transgenic *Drosophila* compared to controls. Further characterization of these differentially methylated RNA isoforms reveal that 353 sites have significantly higher m^6^A modification rates in the tau condition, while 205 have less (Supplemental File 6). As our analysis is dependent on DRACH k-mer motifs, we then plotted the k-mer motif that is altered in the context of tau pathology. The k-mer AAACT followed by GGACA has the most hypermethylated sites, while AAACT, GAACA and AAACA have the most hypomethylated sites (Supplemental Fig. 6A). Differential m^6^A modification occurs predominantly in the coding sequence and 3’ UTR in tau transgenic *Drosophila*, including a shift in hypermethylated sites toward the 3’ UTR (Fig. 5C, Supplemental Fig. 6B, C), as previously reported [82]. We detect the same trend for sites that are hypomethylated in the tau condition (Supplemental Fig. 6B, C). A small subset of transcripts (< 3%) exhibit both hypermethylation and hypomethylation of sites within the same isoform in tau transgenic *Drosophila* compared to controls (Supplemental File 6). A gene ontology analysis of differentially methylated genes reveals that transcripts that are hypomethylated in brains of tau transgenic *Drosophila* are involved in receptor tyrosine kinase signaling and cellular homeostasis (Supplemental Fig. 6D), while those that are hypermethylated are involved in metabolism pathways, including carbohydrate, lipid, and macromolecule metabolic processes that are particularly important for the brain (Supplemental Fig. 6E). No significant correlation (p value = 0.15) is observed between m^6^A modification rate change and isoform expression fold-change (Supplemental Fig. 6F).

While previous studies have identified retrotransposon transcripts that are differentially expressed between control and tau transgenic *Drosophila* [75, 76]*,* it was not previously feasible to identify the source loci for such elements due to the limitations of Illumina sequencing. Using ONT-based differential expression analysis, we identify six retrotransposon source loci that are differentially expressed in tau transgenic *Drosophila* versus controls (Fig. 5D). Among the three loci that are significantly upregulated in tau transgenic *Drosophila* compared to controls, two are *copia* elements present on the 3R chromosome (FlyBase ID: copia 3R: 2,254,785–2,259,872) and chromosome Y (FlyBase ID: copia Y: 1,217,291–1,220,889) (Fig. 5D, Supplemental File 7).

We identify four transposable element loci within the copia family that produce transcripts with differential m^6^A modification (Supplemental Fig. 7A). We find that hypomethylation of the chromosome band 40F7 *copia* element correlates with decreased expression in tauopathy, whereas the *copia* element on chromosome band 4b4-4b5 is hypermethylated and increased at the RNA level (Supplemental Fig. 7B, Supplemental File 7). Taken together, our findings suggest that tau-induced polyA tail lengthening and m^6^A changes significantly contribute to the head transcriptome in *Drosophila*, and tau-induced m^6^A changes may contribute to transposable element dysregulation at the RNA level.

## Discussion

To our knowledge, this study is the first to analyze full-length native RNA using long-read sequencing in heads of a *Drosophila* model of tauopathy. Our work builds upon previous studies by identifying RNA base modifications at the isoform level and identifying locus-specific expression of highly repetitive transposable elements in physiological and pathological settings using a single long-read sequencing strategy that reduces technical variation introduced by weaving together mixed methods [59]. A recent analysis combined sequencing replicates from multiple long-read sequencing methodologies, including ONT cDNA sequencing and direct RNA sequencing, to assemble a full-length *Drosophila* head transcriptome [59], but did not perform analyses of RNA methylation or transposable elements; by assembling a combined transcriptome from head, ovaries and embryos, this previous analysis also lacks *Drosophila* head-specific structural and splicing classification of novel isoforms. In the current study, we analyze head-specific gene expression based on the GAL4/UAS system [73] rather than comparing the head and ovaries [59], which are two functionally distinct tissues that are not equivalent in terms of complexity. A long read derived transcriptome thus closes gaps left by previous long-read sequencing studies and standard Illumina RNA sequencing in *Drosophila* heads and improves our understanding of how post-transcriptional control of gene expression contributes to the tau transcriptome.

We first characterized the *Drosophila* post-transcriptional modifications that had been previously described using alternative methods, with the advantage that our analysis is at isoform level rather than gene level. For example, isoform-level polyA tail profiling [42, 65] and gene ontology analysis report that transcripts with specific biological process are enriched towards certain polyA tail lengths. We also determined the modification status of single transcripts and confirm previous m^6^A-immunoprecipitation sequencing studies in *Drosophila* head [83] and larval brain [68] showing a significant m^6^A enrichment in the 5’ UTR. We did not find an association between m^6^A modification frequency and transcript expression, suggesting that the interaction between m^6^A and transcript expression is more complicated and likely relies on methylation reader proteins in *Drosophila* heads.

An exciting potential of nanopore direct RNA sequencing is its application to transposon biology. Short-read sequencing analyses of transposable element transcriptomes are hampered by technical limitations of aligning repetitive transposable element-derived reads to their loci of origin. Transposable element/gene hybrids such as chimeric transcripts, transposable elements within introns of protein-coding genes, and read-through transcripts contribute further to misalignments and inflated quantification of transposable element-derived transcripts. Prior to the onset of long-read sequencing, most analyses focused only on transposable elements at the family level rather than the locus level. Our analysis identifies multiple *copia* loci as the most highly expressed transposable elements in *Drosophila* heads with a large degree of consensus m^6^A modifications. We identified abundant expression of *copia* isoforms previously shown to encode gag, a protein that is central to the formation of viral-like capsids [84], and mapped the source loci of several transposable element transcripts potentially relevant to *Drosophila* brain biology. We did not detect any chimeric or read-through transposable element-containing transcripts, presumably due to polyA selection and relatively low long-read sequencing coverage.

We next applied nanopore direct RNA sequencing to *Drosophila* with panneuronal expression of human mutant tau. Accumulating evidence links pathogenic forms of tau to aberrant splicing [14, 15, 79, 85], transposable element activation [75, 76, 86], dynamic polyA tail lengthening [87], and altered m^6^A status of RNA [88]. We find that expression of pathogenic tau supports the synthesis of longer polyA tails and elevates m^6^A modification. Comparative analysis of polyA tail length in neurons vs. glia confirmed lengthening of polyA tails for transcripts associated with neurons. The longer polyA tails did not correlate with transcript abundance in tau transgenic *Drosophila* compared to controls. Our dataset thus highlights the technical limitations of ONT polyA tail profiling including a bias toward transcripts with longer polyA tails and difficulty analyzing cell-specific transcripts at low sequencing depths. As expected, we observed significant post-transcriptional alteration of genes in metabolic, host defense- and immune-related biological processes that are also perturbed in mouse and human tauopathy [29, 89–93]. These findings are consistent with a prior single-cell RNA sequencing analysis in *Drosophila* overexpressing human mutant tau^R406W ^[94].

An advantage of nanopore-based m^6^A analysis is the availability of tools with single base level m^6^A calling and quantitative comparisons without the need for a negative control such as a *Drosophila* harboring a loss-of-function mutation in an m^6^A writer. TandemMod and m6Anet are computational methods that use one input sample to predict modifications based on a deep learning framework developed using synthetic and labeled datasets. TandemMod identifies individual feature alterations caused by modified bases and provides prediction models trained on direct RNA sequencing datasets derived from in vitro transcribed sequences, a rice cDNA library, and yeast rRNA [69]. m6Anet training data consisted of labels (modified/unmodified) obtained from m6A-Crosslinking-Exonuclease-sequencing (m6ACE-seq) of HEK293T cell lines and direct RNA sequencing data from HCT116 cell lines [48]. Differences in training datasets may account for different site-level predictions by TandemMod vs. m6Anet, as m^6^A analysis tools developed for nanopore data perform best in the species in which they were trained. Furthermore, TandemMod employs a probability cutoff strategy that results in reduced performance with current signals from mRNA with lower sequencing depth and modification rates as in our dataset.

While the lack of a negative control for nanopore-based m^6^A analysis could result in more false positives, we reduced the potential false positive rate by only considering m^6^A sites that are 1) enriched at the 5’-DRACH-3’ sequence motif, and 2) demonstrated a significant methylation rate change (> 0.4) between control and tau transgenic *Drosophila*. Similar to a recent study using miCLIP [95] to map m^6^A sites in *Drosophila* [52], we find that m^6^A sites are preferentially deposited in 5’ UTRs. As 5’ UTR m^6^A sites can enhance translation and protein output [52, 96–98], and transgenic expression of pathogenic tau results in an increase in RNA methylation and translation in *Drosophila* [99], we speculate that enhanced 5’UTR m^6^A deposition contributes to the global elevation of mRNA translation in tau transgenic *Drosophila*. We also detected a general increase in m^6^A modification of *copia* elements. Given new findings that an essential m^6^A modification in the 5’ UTR regulates retrotransposon mobility and protein expression of long interspersed element-1 (LINE-1) [100], we speculate that a tau-mediated increase in m^6^A RNA methylation could contribute to the retrotransposon activation observed in human and laboratory models of tauopathy [75, 76, 86, 101–103]. Future studies leveraging nanopore direct RNA sequencing for deep sequencing of *Drosophila* transcriptomes may provide additional insights into the role of m^6^A modification as a regulator of transposable element pathogenicity.

While nanopore direct RNA sequencing has been performed in human cell lines [104–106], yeast [21, 107], plant [108], bacteria [109], viruses [110–112], frog embryos and adult mouse brain [106], sequencing RNA up to 26,000 nt in length [110], additional studies are needed to validate the software tools developed for nanopore direct RNA sequencing data analysis and improve the power of this relatively new, state-of-the-art sequencing technique. For example, we find that using ONT direct RNA sequencing avoids the production of reads with incorrect 3’ ends by reverse transcription mispriming, but leads to transcript read lengths significantly shorter than long (> 10 kb) transcripts typical of the complex nervous system [9, 10]. We also find instances of intron retention and alternative splicing combinations in tau transgenic *Drosophila*, but the effect size is relatively small compared to previous reports using alternative sequencing techniques [14, 79, 85, 113]. We find that site-level precision of m^6^A analysis tools trained on different nanopore datasets [48, 69] significantly varies in low-depth sequencing samples including modification sites with low modification rate. These results may be due to the low sequencing depth of 1–2 million reads of our nanopore-based analysis versus 20–40 million reads typical for traditional amplification-based RNA sequencing methods [114]. The low number of aligned reads per flow cell in the MinION hindered efforts to create de novo transcript assemblies. Some transposable elements, including short interspersed nuclear elements, are not polyadenylated and were thus not sequenced due to the polyA enrichment step that we applied prior to sequencing. The 3’ sequencing bias of nanopore direct RNA sequencing [115, 116] has also been reported to negatively influence isoform detection in *Drosophila* heads [59]. We speculate that the percentage of TSS/PAS-verified full-length reads in our study is masked by decreased basecalling quality and increased basecalling error at mRNA 5′ and 3′ ends [117] that hinder TSS/PAS identification rather than a true deficiency of full-length reads in control and tau transgenic *Drosophila*. New ONT methods have been developed to sequence capped ends of RNA transcripts [21], including RNA transcripts containing repetitive sequences of transposable elements [118], and RNA with or without polyA tails [119].

## Conclusion

The current study identifies physiological and pathological changes in RNA processing that may contribute to age-related neurodegenerative diseases such as Alzheimer’s disease and related tauopathies. We discover that panneuronal expression of pathogenic tau increases polyA tail length, m^6^A modification frequency, and transposable element expression. Nanopore direct RNA sequencing-based detection of full-length transcripts and single base modifications allowed for rapid interrogation of tau-based changes that are missed using Illumina short-read or cDNA sequencing approaches. Our datasets thus provide new insights into the impact of pathological tau on the transcriptome and establishes a framework for future studies characterizing the complex tau transcriptome in humans.

## Supplementary Information

Supplementary Material 1: Supplemental File 1 **| **Excel file of m^6^A-modified genes in adult *Drosophila* heads compared to target genes identified as m^6^A-modified from m^6^A RIP-seq-based analysis of the *Drosophila* larval brain.

Supplementary Material 2: Supplemental File 2** | **Excel file of m^6^A modification analysis using m6Anet vs TandemMod.

Supplementary Material 3: Supplemental File 3** | **Excel file of m6Anet sites and miCLIP site/peak overlap.

Supplementary Material 4: Supplemental File 4** | **Excel file of StringTie transcriptome for Illumina and Nanopore assemblies.

Supplementary Material 5: Supplemental File 5** | **Excel file of polyA tail lengths of *Drosophila* reference transcripts and differential polyA tail length analysis of tau vs control.

Supplementary Material 6: Supplemental File 6** | **Excel file of methylation modifications of *Drosophila* reference transcriptsand differential m^6^A modification analysis of tau vs control.

Supplementary Material 7: Supplemental File 7** | **Excel file of transcript and m^6^A quantification of transposable elements. Differential expression and m^6^A modification analysis of tau vs control transposable elements.

Supplementary Material 8: Supplemental Figure 1 | Comparison of nanopore DRS and Illumina short-read StringTie assemblies. A) Scatterplot of TPM values of short-read isoforms vs. TPM values of long read isoforms. B) Density plot of assembled isoform lengths with five TPM or more.

Supplementary Material 9: Supplemental Figure 2 | Known and novel isoforms identified by SQANTI. Graphic of structural categories and subcategories detected by SQANTI.

Supplementary Material 10: Supplemental Figure 3 | Comparing alternative splicing events contributing to the *Drosophila *transcriptome derived from Illumina short-read sequencing and nanopore direct RNA sequencing. A) Scatterplot of FlyBase reference isoform length vs. nanopore direct RNA sequencing assisted De novo assembly isoform length. The percentage of (B) different structural categories considered novel and (C) their splicing subcategories in the transcriptome derived from Illumina short-read RNA sequencing and nanopore direct RNA sequencing. Direct RNA sequencing data was analyzed using the Guppy or Dorado basecaller. D) Scatter plot of nanopolish and tailfindr polyA tail length values for Control 3 and the associated Pearson correlation value. E) Metagene plot showing the distribution of m^6^A sites in pooled control sample set and miCLIP dataset (single m^6^A sites) of adult fly heads.

Supplementary Material 11: Supplemental Figure 4 | Canonical transcript isoforms are differentially expressed in heads of tau transgenic *Drosophila* compared to controls. A) PCA plot of expression levels generated by nanopore direct RNA sequencing in heads of tau transgenic *Drosophila* compared to control. B) Pearson’s correlation plot visualizing correlation values of all isoform TPM values. C) Alternative splicing of Rps21 produces two transcript isoforms that are differentially expressed at the RNA level in tau transgenic *Drosophila*. D) Integrative genomics viewer of enolase isoforms. Violin plot of expression fold changes corresponding to (E) different categories of spliced isoforms or (F) transcripts containing PTC and non PTC-containing, ‘normal’ transcripts in tau transgenic *Drosophila* compared to control. Using the (G) Guppy or (H) Dorado basecaller, we determined the percentage of different structural categories from pooled control and pooled tau STRINGTIE as assemblies. A Fisher’s exact test was used to determine a significant association between intron retention transcripts and fly genotype.

Supplementary Material 12: Supplemental Figure 5 | Expression of pathogenic tau causes a shift in isoform-specific polyA tail length. Distribution plot of isoform-specific RNA polyA tail lengths in (A) control and (B) tau transgenic *Drosophila*. Density graph with (C) short, medium and long poly(A) tail lengths and (D) low, medium, and high expression per read for six different types of transcripts. E) Dot plot of enriched gene ontology terms for RNA transcript isoforms with a significant change in polyA tail length between conditions. F) Scatter plot of isoform transcripts with a significant change in both isoform expression and polyA tail length in tau transgenic *Drosophila* compared to controls. Pearson correlation analysis reveals a significant negative correlation. G) Boxplot of polyA tail lengths of cell marker gene per control and tau sample. Only samples with detected polyA tails for the plotted isoform are shown. H) Scatter plot of isoform transcripts with a significant change in both polyA tail length and m^6^A methylation change in in tau transgenic *Drosophila* compared to controls, with no significant correlation.

Supplementary Material 13: Supplemental Figure 6 | m^6^A methylation patterns are significantly altered in tauopathy. A) Bar graph of differentially methylated DRACH sites grouped by kmer site in tau transgenic *Drosophila* compared to controls. B) Metagene plot showing the distribution of m^6^A sites in pooled tau and pooled control samples. C) Differentially methylated RNA in tau transgenic *Drosophila* preferentially accumulate m^6^A modifications within the coding sequence and 3’ UTR. Dot plot of enriched gene ontology terms for differentially m^6^A modified isoforms, including (D) hypermethylated and (E) hypomethylated sites. F) Scatter plot of isoform transcripts with a significant change in both isoform expression and m^6^A methylation change in tau transgenic *Drosophila* compared to controls, with no significant correlation.

Supplementary Material 14: Supplemental Figure 7 | Nanopore direct RNA sequencing identifies transposable elements at the loci level that are differentially methylated. A) Scatter plot of transposable element loci identifies a subset that are hypermethylated and more highly expressed at the RNA level in tauopathy. B) The transposable element, *copia*, is differentially methylated at specific loci in the *Drosophila* genome.

## Data Availability

The data generated and/or analyzed during the current study will be deposited within the GEO repository, GSE305884. The code for all steps used to create transcriptomes and figures is available at: https://doi.org/10.5281/zenodo.15603223.

## Abbreviations

ONT: Oxford Nanopore Technologies
UTR: Untranslated region
SQANTI: Structural and Quality Annotation of Novel Transcript Isoforms
FLAIR: Full-length Alternative Isoform analysis of RNA
PTC: Premature termination codon
m^6^A: N6-methyladenosine
FDR: False discovery rate
FSM: Full splice match
ISM: Incomplete splice match
NIR: Novel transcript in reference transcriptome
NNR: Novel transcripts not in reference transcriptome
TPM: Transcripts per million
RIP: RNA immunoprecipitation
LTR: Long terminal repeat
AMP-AD: Accelerating Medicines Partnership in Alzheimer’s Disease
LINE-1: Long interspersed element-1
