# Supplementary figures and images for: Long-read sequencing-based analyses of the adult Drosophila brain transcriptome in physiological and pathological settings
Source: BMC Genomics. 2025 Oct 14;26:913. doi: 10.1186/s12864-025-12111-w (PMC12522962; doi:10.1186/s12864-025-12111-w)

A

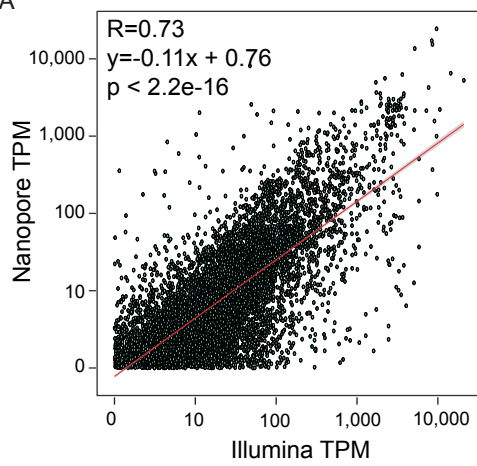

B

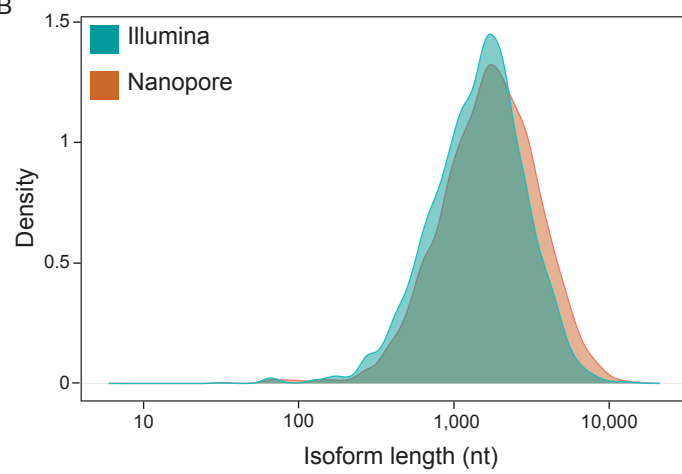

Supplement: Supplementary file 8 — Supplementary Material 8: Supplemental Figure 1 | Comparison of nanopore DRS and Illumina short-read StringTie assemblies. A) Scatterplot of TPM values of short-read isoforms vs. TPM values of long read isoforms. B) Density plot of assembled isoform lengths with five TPM or more. [file 12864_2025_12111_MOESM8_ESM.pdf]

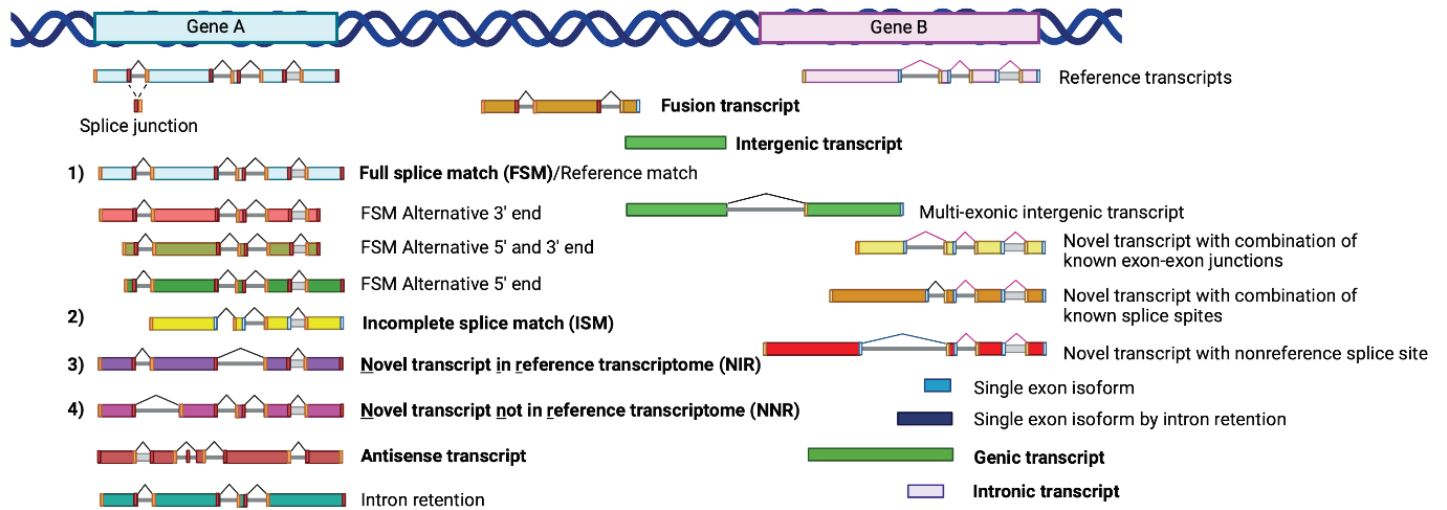

Supplement: Supplementary file 9 — Supplementary Material 9: Supplemental Figure 2 | Known and novel isoforms identified by SQANTI. Graphic of structural categories and subcategories detected by SQANTI. [file 12864_2025_12111_MOESM9_ESM.pdf]

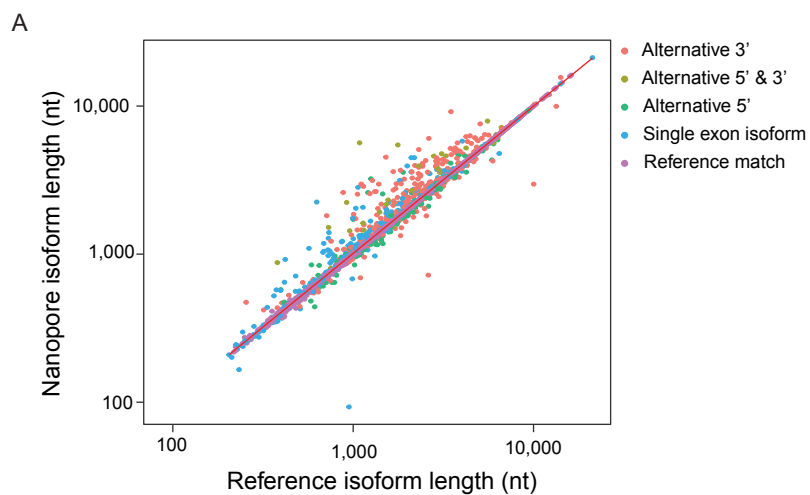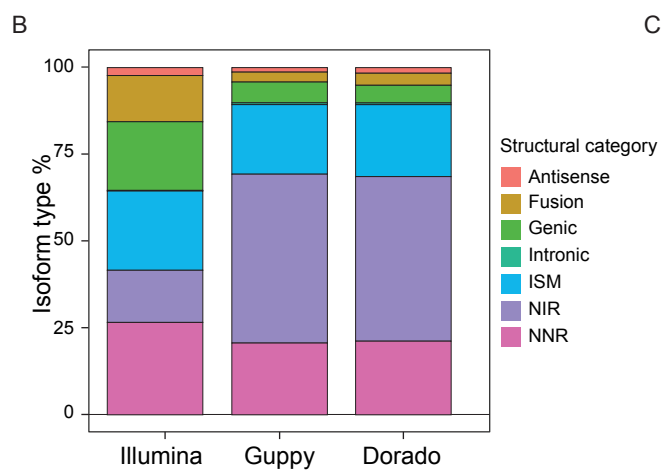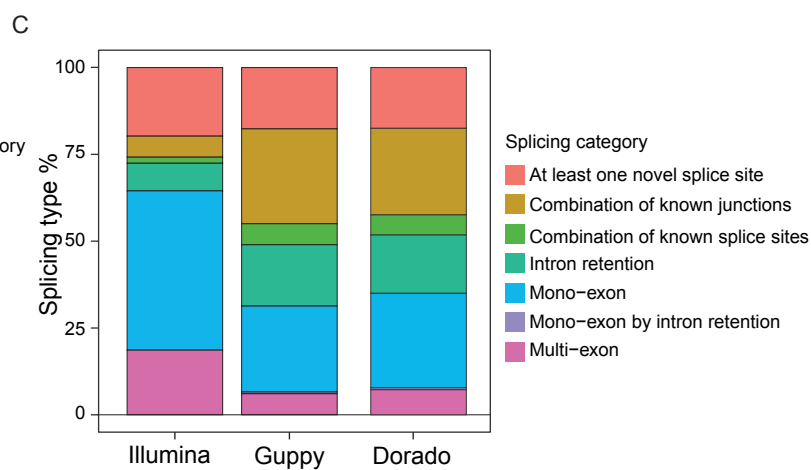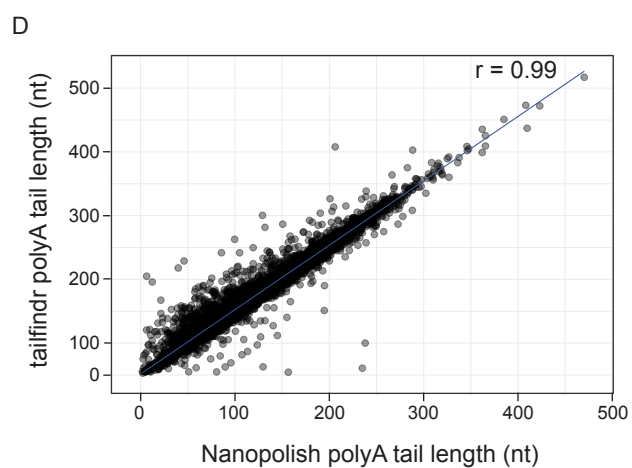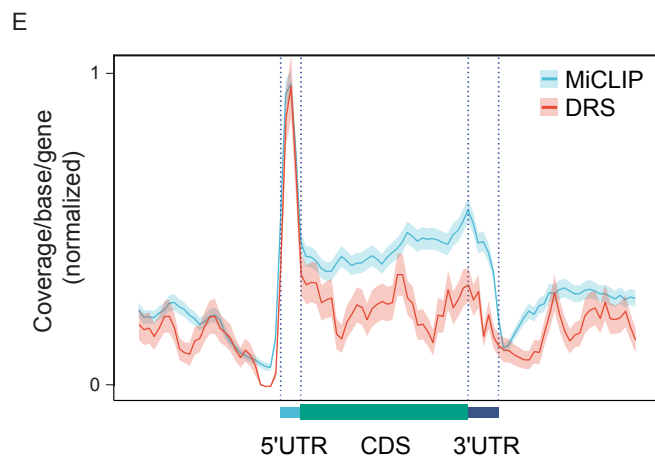

Supplement: Supplementary file 10 — Supplementary Material 10: Supplemental Figure 3 | Comparing alternative splicing events contributing to the Drosophila transcriptome derived from Illumina short-read sequencing and nanopore direct RNA sequencing. A) Scatterplot of FlyBase reference isoform length vs. nanopore direct RNA sequencing assisted De novo assembly isoform length. The percentage of (B) different structural categories considered novel and (C) their splicing subcategories in the transcriptome derived from Illumina short-read RNA sequencing and nanopore direct RNA sequencing. Direct RNA sequencing data was analyzed using the Guppy or Dorado basecaller. D) Scatter plot of nanopolish and tailfindr polyA tail length values for Control 3 and the associated Pearson correlation value. E) Metagene plot showing the distribution of m6A sites in pooled control sample set and miCLIP dataset (single m6A sites) of adult fly heads. [file 12864_2025_12111_MOESM10_ESM.pdf]

**A** Principal Component Analysis (PCA)

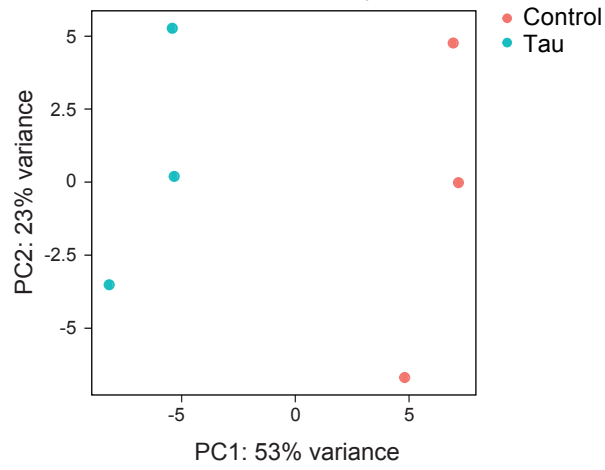

**B**

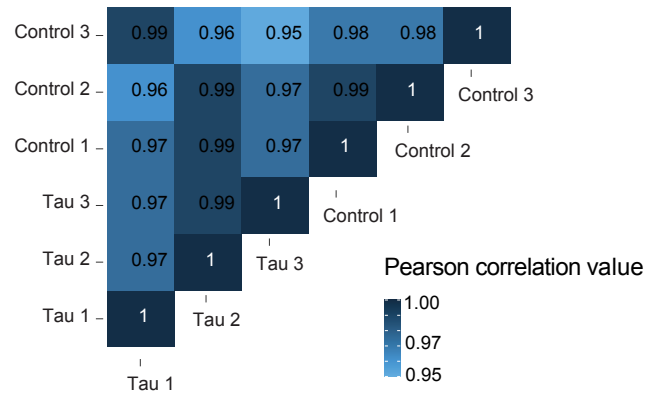

**C**

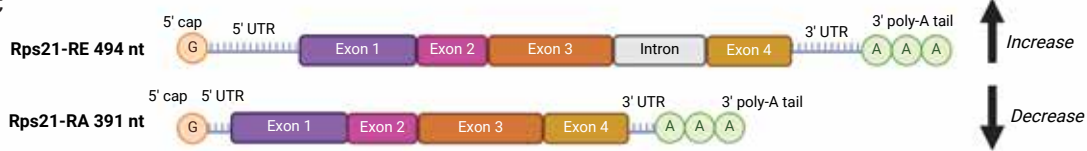

**D**

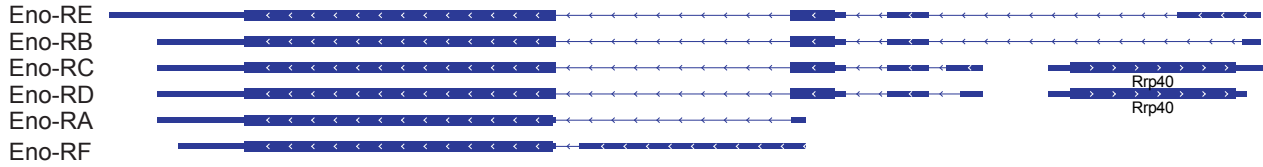

**E**

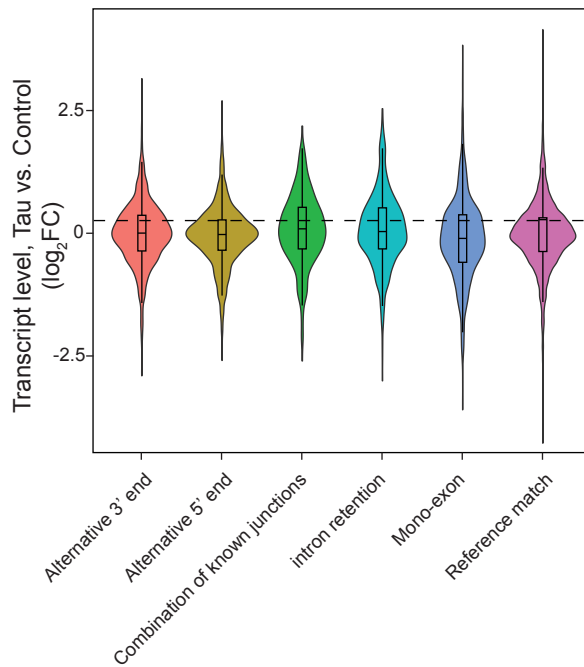

**F**

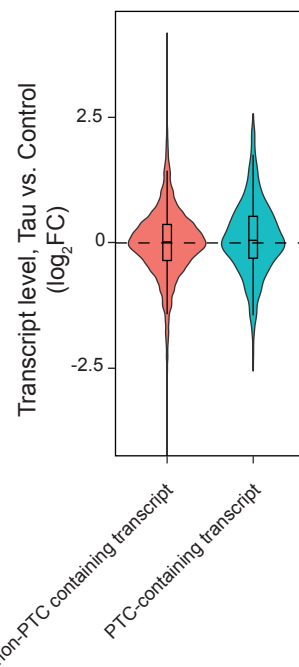

**G**

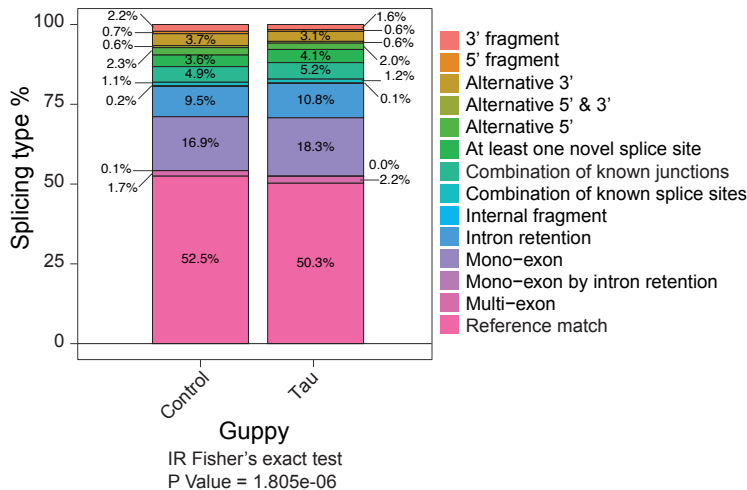

**H**

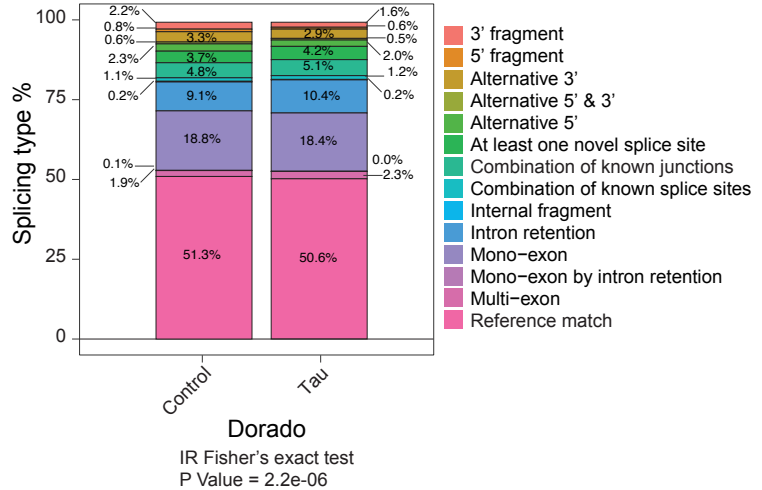

Supplement: Supplementary file 11 — Supplementary Material 11: Supplemental Figure 4 | Canonical transcript isoforms are differentially expressed in heads of tau transgenic Drosophila compared to controls. A) PCA plot of expression levels generated by nanopore direct RNA sequencing in heads of tau transgenic Drosophila compared to control. B) Pearson’s correlation plot visualizing correlation values of all isoform TPM values. C) Alternative splicing of Rps21 produces two transcript isoforms that are differentially expressed at the RNA level in tau transgenic Drosophila. D) Integrative genomics viewer of enolase isoforms. Violin plot of expression fold changes corresponding to (E) different categories of spliced isoforms or (F) transcripts containing PTC and non PTC-containing, ‘normal’ transcripts in tau transgenic Drosophila compared to control. Using the (G) Guppy or (H) Dorado basecaller, we determined the percentage of different structural categories from pooled control and pooled tau STRINGTIE as assemblies. A Fisher’s exact test was used to determine a significant association between intron retention transcripts and fly genotype. [file 12864_2025_12111_MOESM11_ESM.pdf]

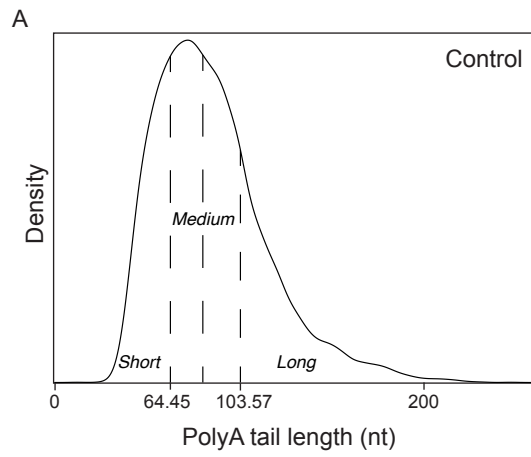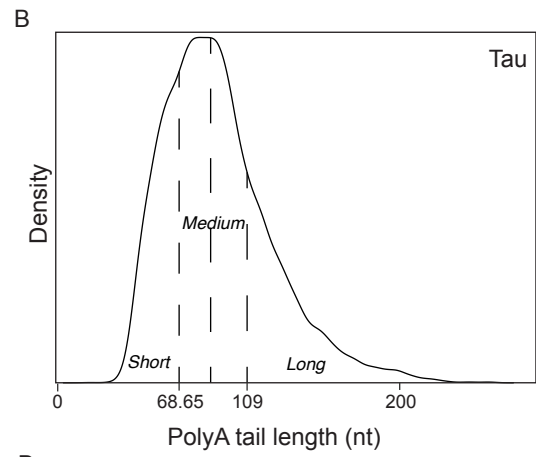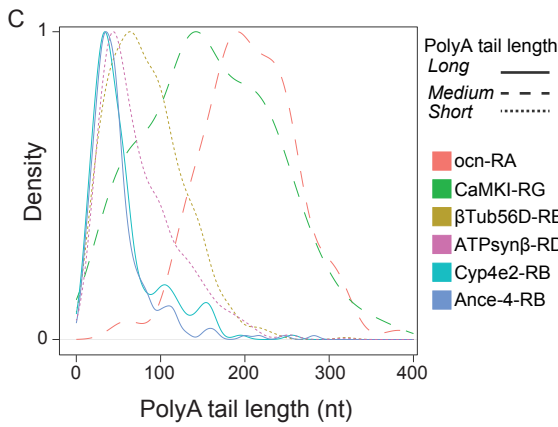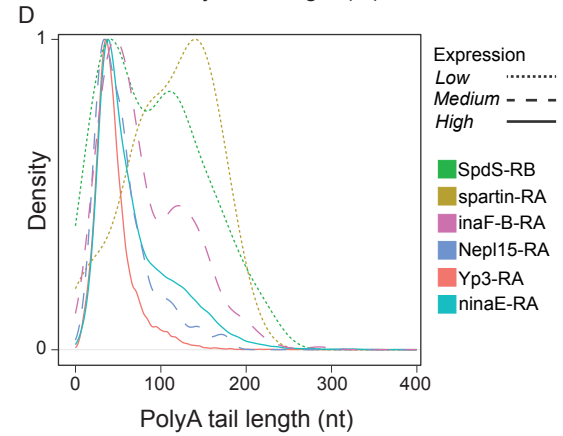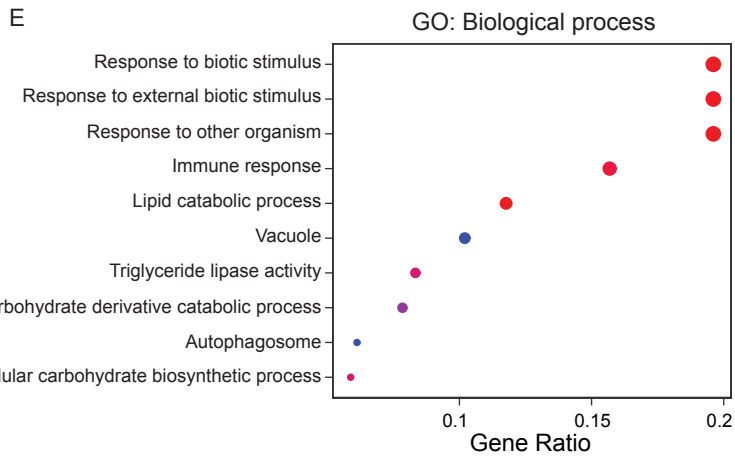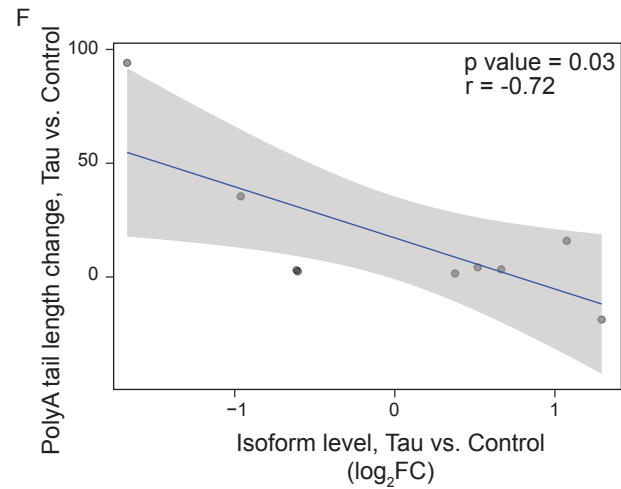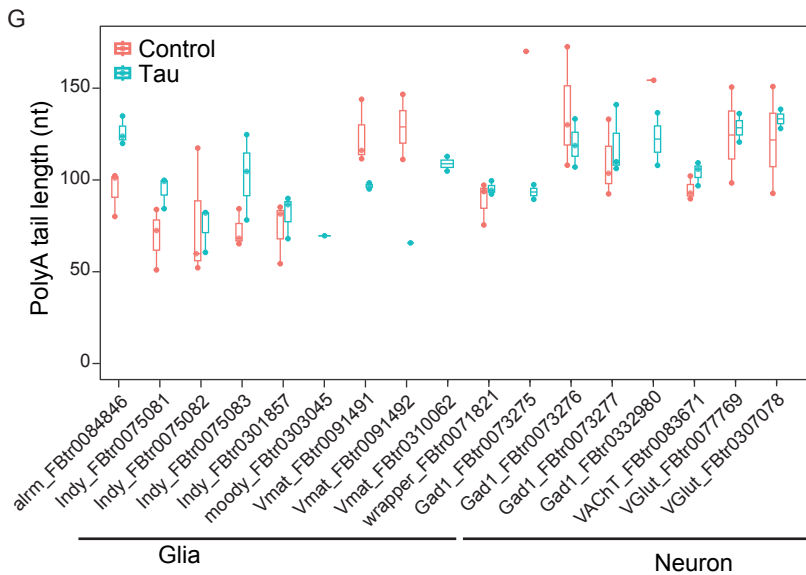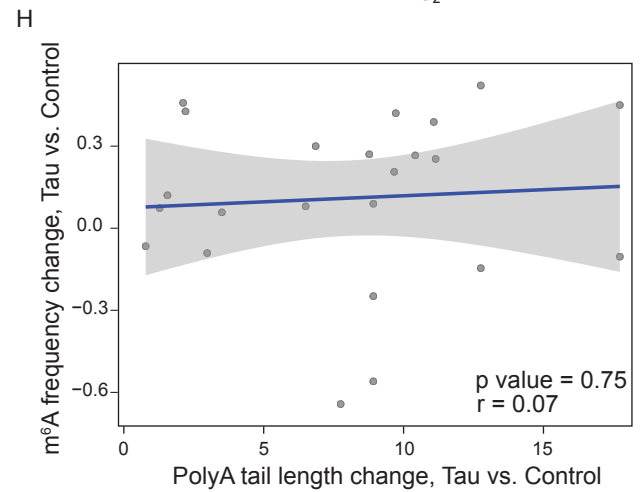

Supplement: Supplementary file 12 — Supplementary Material 12: Supplemental Figure 5 | Expression of pathogenic tau causes a shift in isoform-specific polyA tail length. Distribution plot of isoform-specific RNA polyA tail lengths in (A) control and (B) tau transgenic Drosophila. Density graph with (C) short, medium and long poly(A) tail lengths and (D) low, medium, and high expression per read for six different types of transcripts. E) Dot plot of enriched gene ontology terms for RNA transcript isoforms with a significant change in polyA tail length between conditions. F) Scatter plot of isoform transcripts with a significant change in both isoform expression and polyA tail length in tau transgenic Drosophila compared to controls. Pearson correlation analysis reveals a significant negative correlation. G) Boxplot of polyA tail lengths of cell marker gene per control and tau sample. Only samples with detected polyA tails for the plotted isoform are shown. H) Scatter plot of isoform transcripts with a significant change in both polyA tail length and m6A methylation change in in tau transgenic Drosophila compared to controls, with no significant correlation. [file 12864_2025_12111_MOESM12_ESM.pdf]

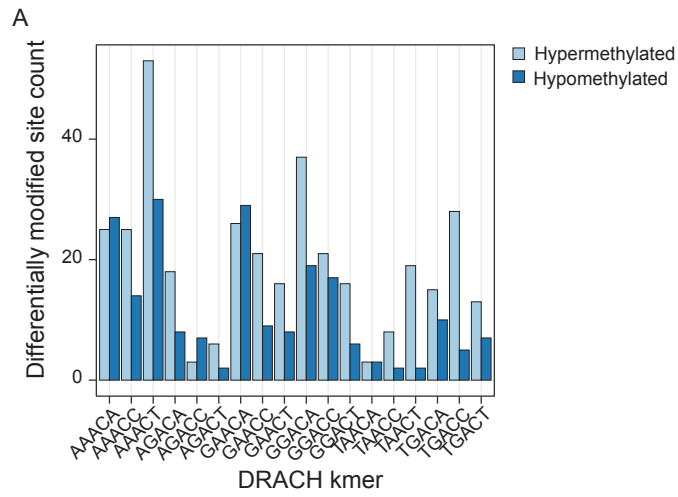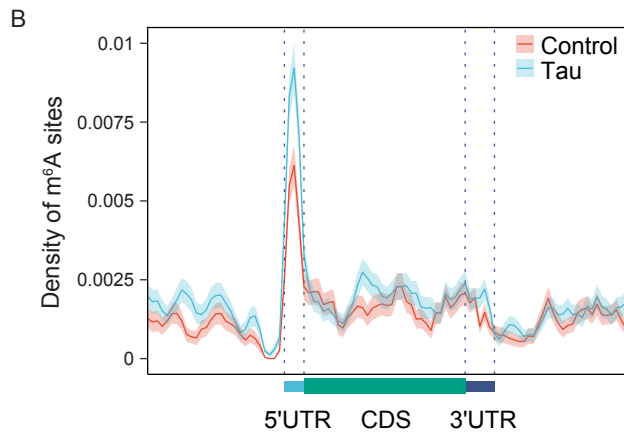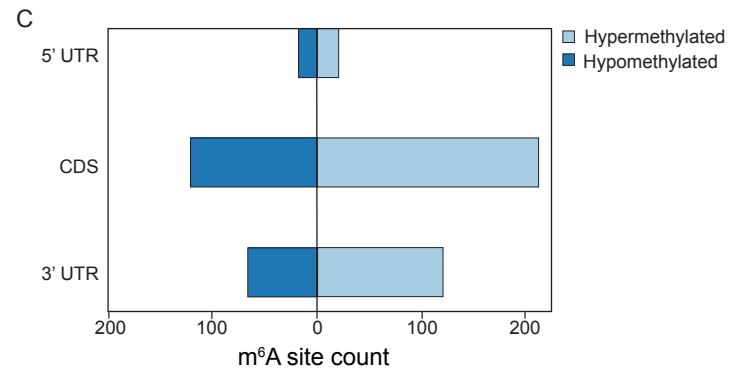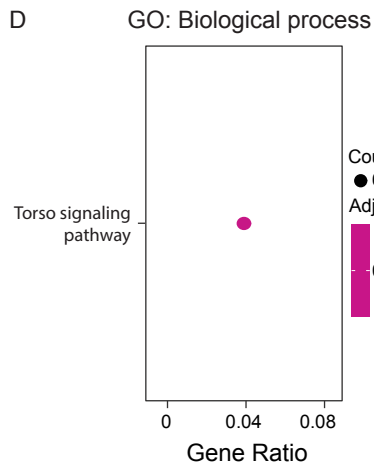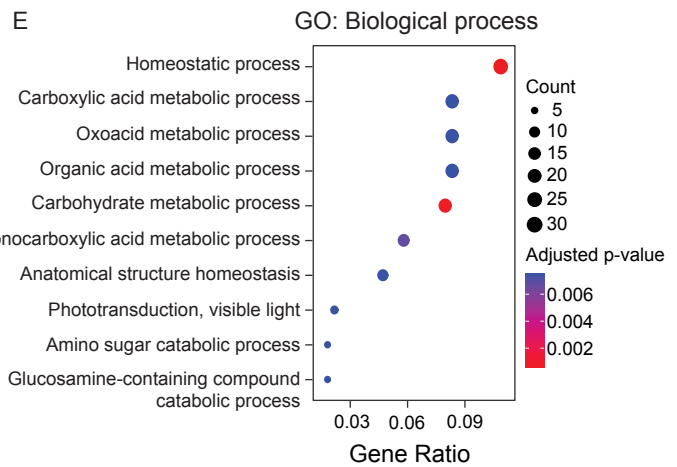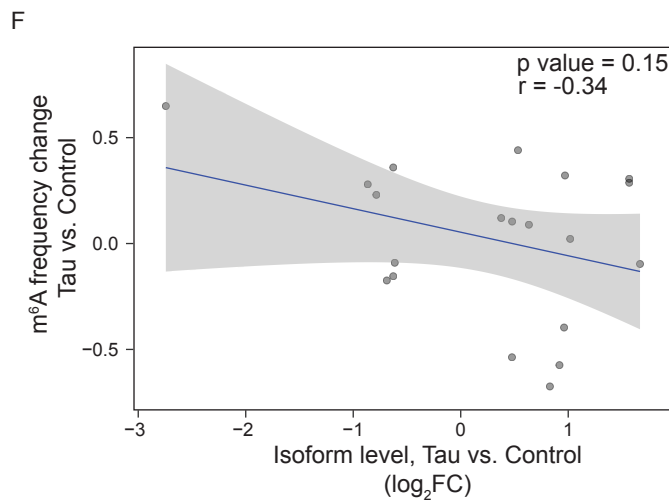

Supplement: Supplementary file 13 — Supplementary Material 13: Supplemental Figure 6 | m6A methylation patterns are significantly altered in tauopathy. A) Bar graph of differentially methylated DRACH sites grouped by kmer site in tau transgenic Drosophila compared to controls. B) Metagene plot showing the distribution of m6A sites in pooled tau and pooled control samples. C) Differentially methylated RNA in tau transgenic Drosophila preferentially accumulate m6A modifications within the coding sequence and 3’ UTR. Dot plot of enriched gene ontology terms for differentially m6A modified isoforms, including (D) hypermethylated and (E) hypomethylated sites. F) Scatter plot of isoform transcripts with a significant change in both isoform expression and m6A methylation change in tau transgenic Drosophila compared to controls, with no significant correlation. [file 12864_2025_12111_MOESM13_ESM.pdf]
