# Supplementary material for: Long-read sequencing-based analyses of the adult Drosophila brain transcriptome in physiological and pathological settings
Source: BMC Genomics. 2025 Oct 14;26:913. doi: 10.1186/s12864-025-12111-w (PMC12522962; doi:10.1186/s12864-025-12111-w)
Supplement: Supplementary file 14 — Supplementary Material 14: Supplemental Figure 7 | Nanopore direct RNA sequencing identifies transposable elements at the loci level that are differentially methylated. A) Scatter plot of transposable element loci identifies a subset that are hypermethylated and more highly expressed at the RNA level in tauopathy. B) The transposable element, copia, is differentially methylated at specific loci in the Drosophila genome. [file 12864_2025_12111_MOESM14_ESM.docx]

|  | **Genes** | **Reference isoforms** | **Non-reference isoforms (mean nt length)** | **# of reads used for assembly** |
| --- | --- | --- | --- | --- |
| Nanopore direct RNA sequencing | 8,223 | 9,179 | 930 (12,604) | 3,035,093 |
| Illumina short-read sequencing | 46,668 | 5,121 | 775 (557) | 223,711,390 |
| FlyBase reference | 50,795 | 72,495 | NA | NA |

**SUPPLEMENTAL TABLES**

**Supplemental Table 1 |** **Comparison of nanopore DRS and Illumina short-read assembly metrics.** StringTie assembly metrics of isoforms (minimum 5 TPM support) compared to FlyBase reference.

| **Isoform Category** | **Illumina** | **Nanopore** | **Fisher P value** |
| --- | --- | --- | --- |
| Anti-sense | 12 (0.20%) | 10 (0.10%) | 0.0828 |
| Reference | 5,121 (87.26%) | 9,179 (90.08%) | >0.0001 |
| Fusion | 70 (1.19%) | 22 (0.22%) | >0.0001 |
| Genic | 104 (1.77%) | 46 (0.46%) | >0.0001 |
| Intronic | 1 (0.02%) | 4 (0.04%) | 0.4376 |
| Reference with different TTS/TSS | 120 (2.04%) | 154 (1.52%) | 0.0144 |
| Intergenic | 222 (3.78%) | 161 (1.59%) | >0.0001 |
| Novel isoform within catalog splice junctions/sites | 79 (1.35%) | 374 (3.70%) | >0.0001 |
| Novel not in catalog splice junctions/sites | 140 (2.39%) | 159 (1.57%) | 0.0003 |

**Supplemental Table 2 | Comparison of nanopore DRS and Illumina short-read isoform splicing.** Percentages and counts of different isoform splice events.

|  | **Tau 1** | **Tau 2** | **Tau 3** |
| --- | --- | --- | --- |
| Total reads sequenced | 966,820 | 1,790,617 | 1,379,790 |
| Longest isoform sequenced (nt) | 23,647 | 32,265 | 22,944 |
| Median read quality | 11.3 | 11.8 | 11.8 |
| Read length (n50) | 1,324 nt | 1,399 nt | 1,418 nt |
| Median fraction of reference transcript covered by each read | 0.92 | 0.90 | 0.93 |
| Percentage of full length reads | 41.1 | 39.2 | 43.1 |
| Mapping percentage to *Dmel* genome | 91.0 | 92.6 | 92.0 |
| TSS/PAS-verified full length reads | 3.33 | 3.27 | 3.40 |

**Supplemental Table 3 |** **Comparison of nanopore DRS metrics across tau samples.** Nanopore direct RNA sequencing quality metrics of sequencing libraries in tau transgenic *Drosophila*.
